# ETHE1 dampens colorectal cancer angiogenesis by promoting TC45 Dephosphorylation of STAT3 to inhibit VEGF-A expression

**DOI:** 10.1038/s41419-024-07021-w

**Published:** 2024-08-28

**Authors:** Xiaowei She, Jialu Xu, Haokun Zhang, Chengxin Yu, Zejun Rao, Jiakun Zhang, Wenli Zhan, Fuqing Hu, Da Song, Haijie Li, Xuelai Luo, Guihua Wang, Junbo Hu, Senyan Lai

**Affiliations:** 1 GI Cancer Research Institute, Tongji Hospital, Huazhong University of Science and Technology, Wuhan, China; 2 Department of Endocrinology, Tongji Hospital, Tongji Medical College, Huazhong University of Science and Technology, Wuhan, China; 3 Tongji Medical College and State Key Laboratory for Diagnosis and Treatment of Severe Zoonotic Infectious Disease, Huazhong University of Science and Technology, Wuhan, Hubei China

**Keywords:** Tumour angiogenesis, Colorectal cancer

## Abstract

Angiogenesis is critical for colorectal cancer (CRC) progression, but its mechanisms remain unclear. Here, we reveal that ethylmalonic encephalopathy protein 1 (ETHE1), an essential enzyme in hydrogen sulfide catabolism, inhibits VEGF-A expression and tumor angiogenesis in vitro and in vivo. Moreover, we find that this biological function of ETHE1 depends on the STAT3/VEGF-A pathway. Further investigation demonstrates that ETHE1 promotes the interaction between T cell protein tyrosine phosphatase (TC45) and STAT3, resulting in decreased STAT3 phosphorylation and inhibition of the STAT3 signaling pathway. In clinical samples, we find that ETHE1 is downregulated in CRC and positively correlates with survival outcomes of CRC patients. Meanwhile, the negative correlation of ETHE1 and VEGF-A expression is verified in CRC specimens, and the patients with low ETHE1 and high VEGF-A expression exhibits poorer prognosis. Collectively, our study identifies ETHE1 as a novel regulator of tumor angiogenesis, implying its potential as a prognostic biomarker and promising antiangiogenic target for CRC patients.

## Introduction

Colorectal cancer (CRC) is a prevalent and malignant disease of the digestive system, a major contributor to cancer-related deaths worldwide [1]. Despite advancements in CRC treatments including surgery, radiotherapy, chemotherapy, targeted therapy, and immunotherapy, the prognosis for CRC patients remains unfavorable [2, 3]. Hence, identifying a sensitive and specific biomarker, and understanding the molecular mechanisms that drive CRC tumorigenesis and progression, holds profound clinical significance.

Angiogenesis, a pivotal hallmark of solid cancer, is a fundamental pathological process that promotes the development and progression of CRC [4, 5]. As the tumor progresses beyond microscopic size, the activation of the “angiogenic switch” tilts the balance of pro- and anti-angiogenic factors directly towards pro-angiogenic stimuli, leading to tumor angiogenesis [6]. Among the identified pro-angiogenic factors, vascular endothelial growth factor A (VEGF-A) is recognized as a major regulator of pathological blood vessel growth and maintenance [7]. Consequently, anti-VEGF-A therapy has garnered considerable attention over the past few decades. Several drugs, such as bevacizumab, have been approved for clinical application and achieved impressive outcomes in CRC [8, 9]. Nevertheless, due to the heterogeneity of tumor patients, cases of poor efficacy or even drug resistance are common, limiting the effectiveness of these drugs [9]. Therefore, it is crucial to explore novel approaches for regulating VEGF-A and identifying additional therapeutic targets in CRC.

Ethylmalonic encephalopathy protein 1 (ETHE1), as a member of non-heme iron enzymes, catalyzes the transformation of sulfur-containing substrates without requiring cofactors, specifically deoxygenating glutathione persulfide (GSSH) into glutathione (GSH) and sulfite [10, 11]. It has been well documented that *ETHE1* is the key gene responsible for a rare autosomal dominant inherited metabolic disease, which is termed Ethylmalonic Encephalopathy [12, 13]. ETHE1 has been confirmed to participate in ferroptosis in hepatocellular carcinoma and in metastasis in breast cancer [14, 15]. Unexpectedly, as a phylogenetically conserved protein, the functional regulation of ETHE1 in CRC angiogenesis has not been elucidated.

In this study, we initially discovered that ETHE1 is downregulated and holds prognostic significance in CRC patients. Subsequently, we aimed to comprehensively investigate the role of ETHE1 in CRC angiogenesis using both in vitro and in vivo models. Furthermore, we identified the mechanisms through which ETHE1 mediates the development of CRC angiogenesis. This study provides novel insights into the mechanisms of colon cancer angiogenesis and identifies ETHE1 as a potential therapeutic target.

## Results

### Low expression of ETHE1 is related to poor prognosis of CRC patients

**Fig. 1:**
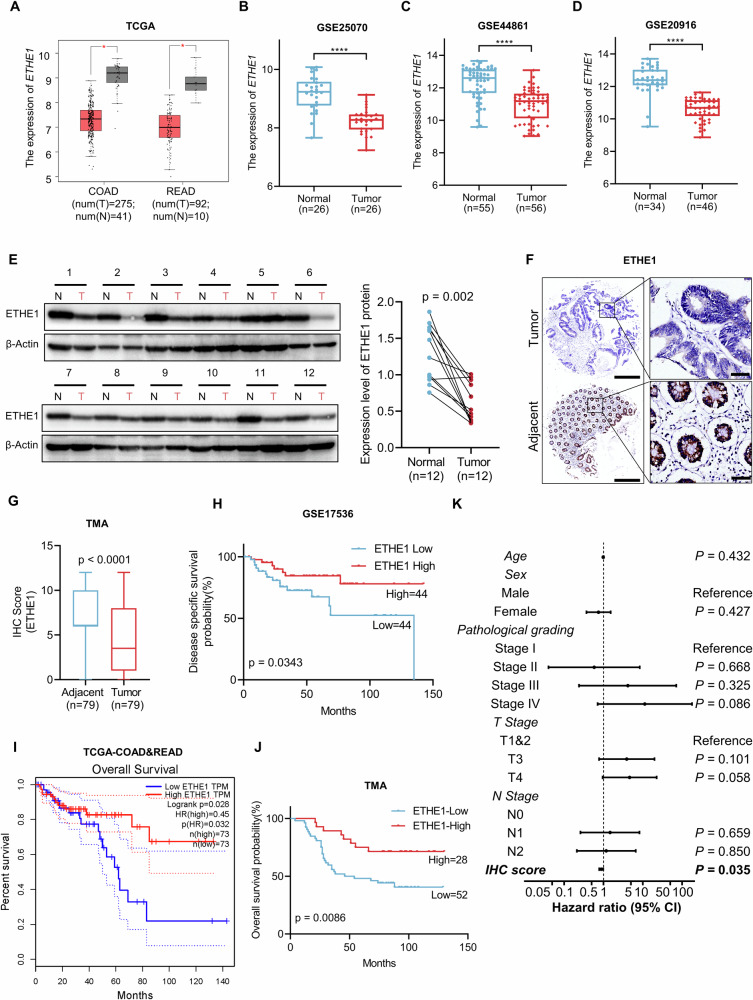
Downregulated ETHE1 is observed in colorectal cancer and associated with a worse prognosis. **A** The mRNA level of *ETHE1* in CRC tissues compared with adjacent normal tissues using the TCGA-COAD and -READ datasets from the GEPIA online tool. Colon adenocarcinoma (COAD), and Rectum adenocarcinoma (READ). Student’s two-tailed test, *p* < 0.05. **B**–**D** The mRNA level of *ETHE1* in CRC tissues compared with adjacent tissues using the GSE25070, GSE44861, and GSE20916 datasets. Student’s two-tailed t test, *p* < 0.001. **E** The protein level of ETHE1 detected by IB assay in 12-paired colorectal tumor and adjacent normal tissues (Left) and quantitative analysis of the 12-paired colorectal tumor and adjacent normal tissues (Right), paired t test, *p* = 0.002. **F** Representative images of ETHE1 expression from the CRC tissues microarray (TMA). Scale bar of left panels = 500 μm and scale bar of right panels = 100 μm. **G** Quantitative analysis of The IHC score of ETHE1 in the TMA. Student’s two-tailed t test, *p* < 0.001. **H** Kaplan–Meier Disease specific survival (DFS) analysis in the GSE17536 dataset according to *ETHE1* expression. Log-rank test, *p* = 0.0343. **I** Kaplan–Meier Overall survival (OS) analysis in the TCGA-COAD and -READ datasets according to *ETHE1* expression from the GEPIA online tool. Log-rank test, *p* = 0.028. **J** Kaplan–Meier Overall survival (OS) analysis according to ETHE1 expression in the TMA. Log-rank test, *p* = 0.0086. **K** Cox regression analysis shows the significance of the relationship between ETHE1 expression and prognosis for patients with CRC in the presence of other clinical variables.

To investigate the role of ETHE1 in CRC progression, we first evaluated the mRNA expression of *ETHE1* in CRC using the public data from The Cancer Genome Atlas (TCGA) and Gene Expression Omnibus (GEO) datasets (GSE25070, GSE44861, GSE20916). The results showed that the mRNA expression of *ETHE1* was significantly reduced in CRC tissues compared with normal tissues (Fig. 1A–D). Next, we detected ETHE1 protein level in 12 pairs of CRC samples and found that 91.7% (11/12) of tumor tissues had lower ETHE1 expression than the adjacent normal tissues (Fig. 1E). We further conducted immunohistochemical staining assays on a CRC tissue microarray using anti-ETHE1 antibody. Consistent with the above results, representative IHC staining and ETHE1 IHC score exhibited a marked decrease of ETHE1 in CRC tissues compared to the adjacent normal tissues (Fig. 1F, G). Furthermore, we analyzed the correlation between the expression levels of ETHE1 and several clinicopathological parameters of patients with CRC (Table 1). The expression level of ETHE1 did not show a significant correlation with age, gender, and M staging, while it was significantly correlated with T staging, N staging, and pathological grading. Using the Gene Expression Profiling Interactive Analysis (GEPIA) databases, GSE17536 and our CRC tissue microarray, Kaplan–Meier analysis showed that lower ETHE1 expression was significantly related to poor survival outcomes among patients with CRC (Fig. 1H–J). Moreover, we carried out Cox regression analysis to assess the significance of ETHE1 for the prognosis of CRC patients (Table 2). The results indicated that ETHE1 expression could serve as an independent and meaningful prognostic factor for patients with CRC, suggesting that lower expression of ETHE1 may promote CRC malignant progression (Fig. 1K). Collectively, our data confirmed that ETHE1 was downregulated in CRC and ETHE1 expression may be a potential independent factor for predicting prognosis of patients with CRC.

**Table 1:** Association of ETHE1 expression with patient characteristics.

Characteristics	ETHE1-Low	ETHE1-High	*P* value
*n*	52	28	
*Age, mean* *±* *sd*	60.519 ± 13.184	60.964 ± 11.628	0.881
*Sex, n (%)*			0.335
Male	22 (27.5%)	15 (18.8%)	
Female	30 (37.5%)	13 (16.2%)	
*Pathological grading, n (%)*			0.044
Stage I&II	23 (28.7%)	19 (23.8%)	
Stage III&IV	29 (36.2%)	9 (11.2%)	
*T stage, n (%)*			0.040
T1&2	11 (13.8%)	8 (10%)	
T3	13 (16.2%)	13 (16.2%)	
T4	28 (35%)	7 (8.8%)	
*N stage, n (%)*			0.018
N0	25 (31.2%)	19 (23.8%)	
N1	11 (13.8%)	8 (10%)	
N2	16 (20%)	1 (1.2%)	
*M stage, n (%)*			0.358
M0	43 (53.8%)	26 (32.5%)	
M1	9 (11.2%)	2 (2.5%)

**Table 2:** Cox multivariate analysis of the correlation between clinicopathological parameters and survival time of individuals with CRC.

Characteristics	Total(n)	HR (95% CI) Multivariate analysis	*P* value
*Age*	80	0.99 (0.96–1.02)	0.432
*Sex*	80		
Male	42	Reference	
Female	38	0.75 (0.37–1.53)	0.427
*Pathological grading*	80		
Stage I	15	Reference	
Stage II	27	0.58 (0.04–8.39)	0.668
Stage III	27	4.16 (0.24–71.08)	0.325
Stage IV	11	11.25 (0.71–177.97)	0.086
*T stage*	80		
T1&2	19	Reference	
T3	26	3.90 (0.77–19.83)	0.101
T4	35	4.62 (0.95–22.48)	0.058
*N stage*	80		
N0	44	Reference	
N1	19	1.47 (0.26–8.22)	0.659
N2	17	1.18 (0.21–6.59)	0.85
*ETHE1 IHC score*	80	0.88 (0.78–0.99)	0.035

### ETHE1 inhibits VEGF-A expression and suppresses tumor angiogenesis in vitro

**Fig. 2:**
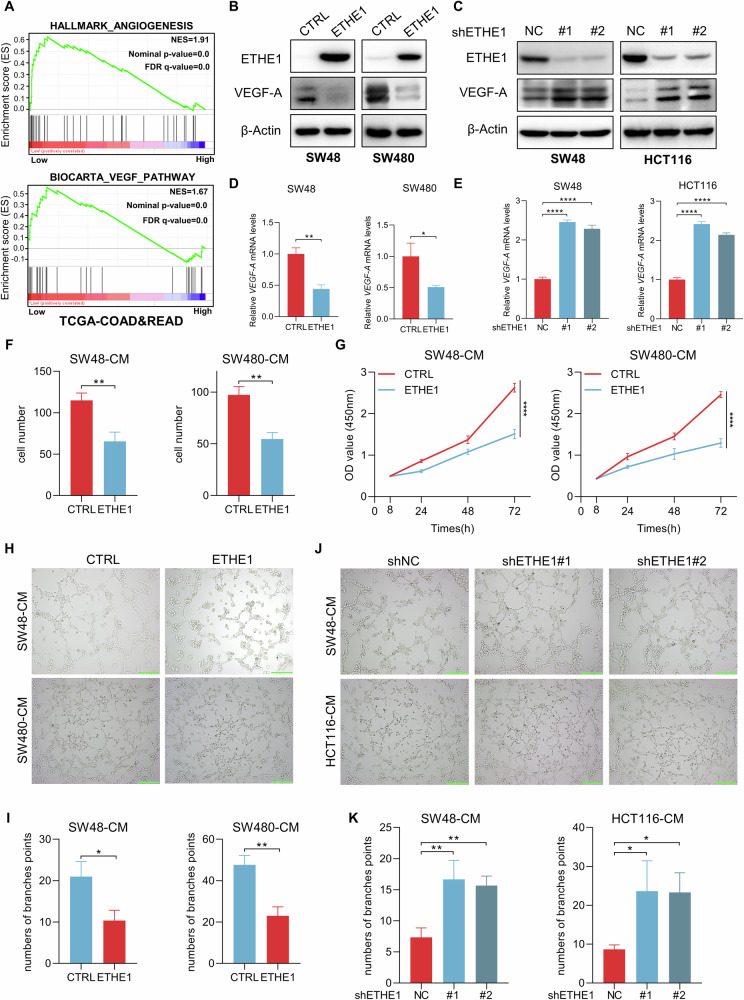
ETHE1 inhibits VEGF-A expression and curbs tumor angiogenesis in vitro. **A** GSEA enrichment plots demonstrate the correlation between the expression of *ETHE1* and Angiogenesis (Up) or VEGF pathway (Down) in the TCGA COAD and READ datasets. **B** The protein level of VEGF-A detected by IB assay in control or ETHE1 stable overexpression SW48 and SW480 cells. **C** The protein level of VEGF-A detected by IB assay in SW48 and HCT116 cells silenced with control (shNC) or ETHE1 shRNA (#1 and #2). **D** The mRNA level of *VEGF-A* detected by qRT-PCR assays in control or ETHE1 stable overexpression SW48 and SW480 cells (*n* = 3). **E** The mRNA level of *VEGF-A* detected by qRT-PCR assays in SW48 and HCT116 cells silenced with control (shNC) or ETHE1 shRNA (#1 and #2) (*n* = 3). **F** Quantitative analysis of Transwell cell migration assay in HUVECs treated with the CM from control or ETHE1 stable overexpression SW48 and SW480 cells (*n* = 3). **G** The viability of HUVECs treated with the CM from control or ETHE1 stable overexpression SW48 and SW480 cells evaluated by using CCK8 assay (*n* = 3). **H**, **J** Representative images of tubule formation assay in HUVECs treated with indicated CM. Green scale bar, 100 μm. **I**, **K** Quantitative analysis of tubule formation from (**H**) and (**I**) (*n* = 3). All immunoblots were performed three times, independently, with similar results. Data are represented as mean ± s.d. *p < 0.05, **p < 0.01, ****p < 0.0001, by (**D**, **F**, **I**) Student’s two-tailed t test and (**E**, **K**) one-way ANOVA with Tukey’s test and (**G**) two-way ANOVA with Tukey’s test.

Given the relevance of ETHE1 expression to the prognosis of CRC patients, our subsequent objective was to identify the underlying biological function of ETHE1. Using a GSEA analysis of TCGA database and GEO datasets (GSE17536, GSE39582), we identified the potential involvement of ETHE1 in angiogenesis and VEGF pathway (Fig. 2A, Supplementary Fig. 1A, B). In addition, the GSEA results highlighted VEGF-A as a core enriched gene. As mounting evidence has implicated that VEGF-A plays a vital role in tumor angiogenesis, we subsequently explored the connection between ETHE1 and VEGF-A. We constructed stable ETHE1-overexpressing SW48 and SW480 CRC cell lines using ETHE1-overexpressing lentivirus and knocked down ETHE1 in SW48 and HCT116 CRC cell lines using lentiviruses containing two distinct shRNA sequences targeting ETHE1, which were validated at both the protein and mRNA levels (Supplementary Fig. 1C–F). As shown in Fig. 2B, C, the expression of *VEGF-A* was reduced with ETHE1 overexpression, whereas increased with ETHE1 silencing. Consistently, the mRNA levels of *VEGF-A* also exhibited a similar change (Fig. 2D, E).

To further explore the effects of ETHE1 on tumor angiogenesis in vitro, we collected the conditional medium (CM) from the indicated CRC cells to stimulate HUVECs. Interestingly, we found that the migration ability of HUVECs was significantly attenuated when incubated with CM from ETHE1-overexpressing SW48 cells compared to incubation with CM from control SW48 cells (Supplementary Fig. 1G, H). In addition, when treated with the CM from ETHE1-overexpressing SW48 and SW480 cells, HUVECs exhibited lower migratory and proliferative abilities (Fig. 2F, G, Supplementary Fig. 1I). However, migration and proliferation of HUVECs were obviously enhanced after incubated with the CM from ETHE1 knockdown SW48 and HCT116 cells (Supplementary Fig. 1J–L). Since HUVECs have the capacity to attach, align, and form capillary-like tubules in Matrigel matrix, tube formation assay represents a well-established model for studying angiogenesis [16]. Intriguingly, HUVECs formed fewer capillary-like tubules when treated with the CM from ETHE1-overexpressing SW48 and SW480 cells (Fig. 2H, I). Furthermore, the CM from SW48 and HCT116 cells with ETHE1 silenced notably bolstered the tube formation ability of HUVECs (Fig. 2J, K). Taken together, these results suggest that decreased expression of ETHE1 in CRC cells promotes VEGF-A expression and tumor angiogenesis in vitro.

### ETHE1 restrains CRC tumor growth and tumor angiogenesis in vivo

**Fig. 3:**
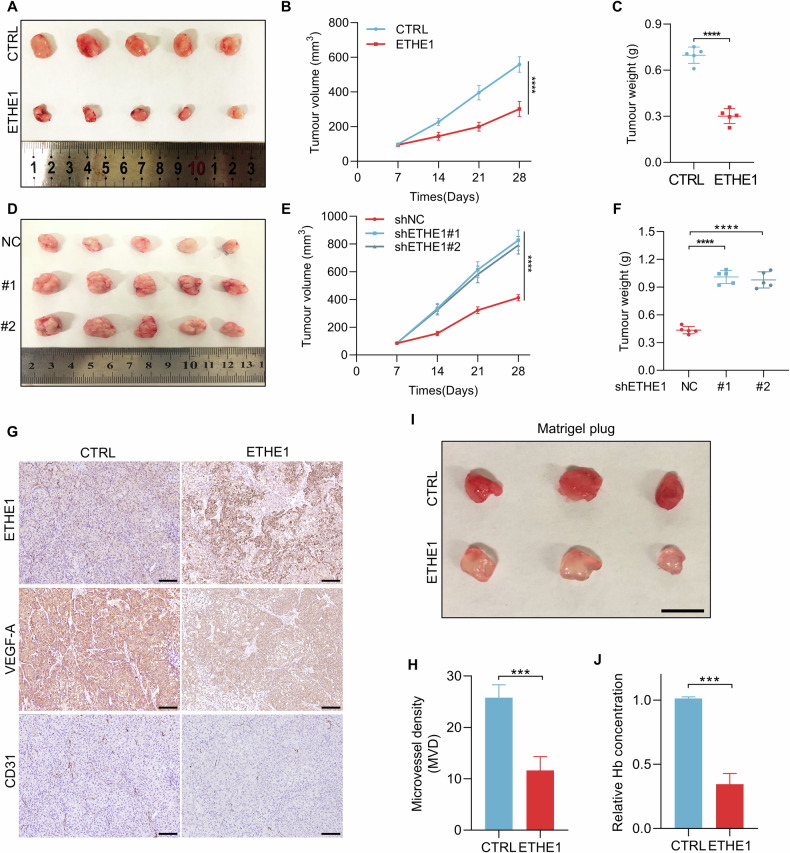
ETHE1 restrains CRC tumor growth and tumor angiogenesis in vivo. **A** Representative image of subcutaneous tumors formed by control or ETHE1 stable overexpression SW480 cells in nude mouse xenograft models. **B**, **C** Quantification of tumor weight and tumor volume generated in (**A**) (*n* = 5). **D** Representative image of subcutaneous tumors formed by SW48 cells silenced with control (shNC) or ETHE1 shRNA (#1 and #2) in nude mouse xenograft models. **E**, **F** Quantification of tumor weight and tumor volume generated in (**D**) (*n* = 5). **G** IHC staining of xenograft tumors generated by control and ETHE1-overexpression SW480 cells using anti-ETHE1, anti-VEGF-A, and anti-CD31 antibodies. Black scale bar, 50 μm. **H** Quantitative analysis of the Microvessel density of subcutaneous tumors according to the staining status of CD31 (*n* = 5). **I** Representative image of the CM-Matrigel plugs formed after 7 days of subcutaneous injection in nude mice. Black scale bar, 1 cm. **J** Quantification of hemoglobin generated in (**I**) (*n* = 3). Data are represented as mean ± s.d. ***p < 0.001, ****p < 0.0001, by (**C**, **H**, **J**) Student’s two-tailed t test and (**F**) one-way ANOVA with Tukey’s test and (**B**, **E**) two-way ANOVA with Tukey’s test.

In vitro experiments have indicated that ETHE1 inhibited VEGF-A expression and suppressed the migration, proliferation, and tube formation of HUVECs. Subsequently, we explored the function of ETHE1 on tumor angiogenesis using subcutaneous xenograft models in vivo. The size of all subcutaneous tumors was monitored every seven days to evaluate tumor growth rate and the mice were sacrificed at 28th days after inoculation. The results showed that overexpressing ETHE1 dramatically restrains tumor growth compared with control group, resulting smaller tumor size (Fig. 3A–C). Conversely, the xenografts derived from SW48 cells with ETHE1 knockdown exhibited faster growth and were heavier than those derived from shNC cells (Fig. 3D–F). Be in congruence with in vitro results, IHC analysis of tumor specimens revealed that the expression of VEGF-A was remarkably reduced in ETHE1-overexpressing tumors (Fig. 3G). In addition, it was note that ETHE1-overexpressing tumors showed abundant CD31 staining, a vascular endothelial cell marker, presenting a high level of microvessel density (Fig. 3G, H). The in vivo angiogenesis plug assay is widely used to evaluate pro- or anti- angiogenic factors [17]. We performed in vivo Matrigel plug assay to further confirm the function of ETHE1 on angiogenesis. The transplant plugs derived from the Matrigel mixed with CM from ETHE1-overexpressing SW48 cells displayed a higher hemoglobin concentration (Fig. 3I, J). In summary, these results indicate that ETHE1 plays a critical role in the suppression of tumor growth and tumor angiogenesis of CRC in vivo.

### ETHE1 impacts the activation of the STAT3 signaling pathway

**Fig. 4:**
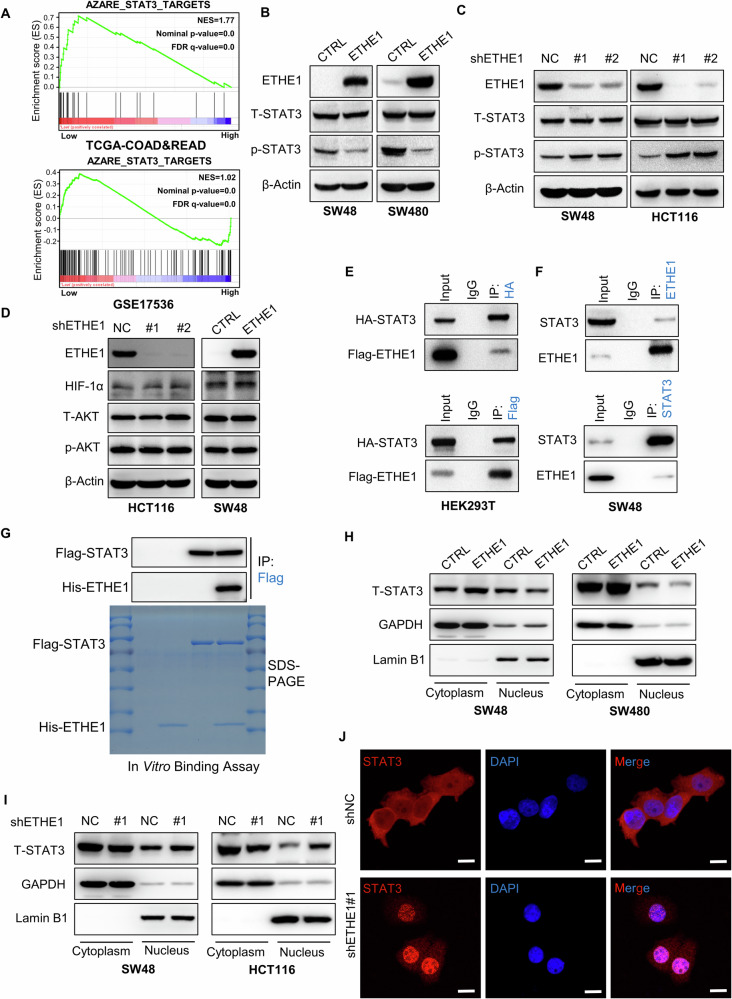
ETHE1 impacts the activation of the STAT3 signaling pathway. **A** GSEA enrichment plots demonstrate the correlation between the expression of ETHE1 and STAT3 Targets in the TCGA COAD and READ datasets (Up) and the GSE17536 dataset (Down). **B** The protein level of T-STAT3 and p-STAT3 detected by IB assay in control or ETHE1 stable overexpression SW48 and SW480 cells. **C** The protein level of T-STAT3 and p-STAT3 detected by IB assay in SW48 and HCT116 cells silenced with control (shNC) or ETHE1 shRNA (#1 and #2). **D** The protein level of HIF-1α, T-AKT and p-AKT detected by IB assay in HCT116 cells silenced with control (shNC) or ETHE1 shRNA (#1 and #2) (Right) and control or ETHE1 stable overexpression SW48 cells (Left). **E** HEK293T cells were transfected with HA-STAT3 and Flag-ETHE1 plasmids, whole cell lysates (WCL) were collected for IP with anti-HA beads or anti-Flag beads, followed by IB analysis. **F** WCL of SW48 cells were collected for IP with anti-STAT3 or anti-ETHE1 antibody, followed by IB analysis. **G** In vitro binding assay was performed. Purified Flag-STAT3 was incubated with His-ETHE1 and pulled down using anti-Flag beads, followed by IB analysis. **H** Cytoplasmic and nuclear proteins were separated from control or ETHE1 stable overexpression SW48 and SW480 cells, followed by IB analysis. **I** Cytoplasmic and nuclear proteins were separated from SW48 and HCT116 cells silenced with control (shNC) or ETHE1 shRNA#1, followed by IB analysis. **J** Representative images of STAT3 subcellular localization observed from SW48 cells silenced with control (shNC) or ETHE1 shRNA#1. White scale bar, 10 μm. All immunoblots were performed three times, independently, with similar results.

Subsequently, we aimed to gain an insight into the molecular mechanisms underlying CRC angiogenesis mediated by ETHE1. Likewise, we performed GSEA analysis and the results showed STAT3-targeted gene signature was significantly elevated among individuals with reduced ETHE1 expression (Fig. 4A). Since the established role of STAT3 pathway in the tumor angiogenesis, we first detected whether ETHE1 could influence the activation of STAT3. As shown in Fig. 4B,C, the western blot assays revealed that overexpression of ETHE1 suppressed phosphorylation of STAT3, whereas knockdown of ETHE1 notably elevated p-STAT3 protein levels. Considering the established role of the Akt signaling pathway and the HIF-1α signaling pathway in tumor angiogenesis, we detected the impact of ETHE1 on these two signaling pathways. The GESA results revealed no correlation between them, and the expression levels of ETHE1 had no effect on the HIF-1α and p-Akt proteins (Supplementary Fig. 2A, B, Fig. 4D). Interesting, when co-transfecting Flag-ETHE1 and HA-STAT3 plasmids into HEK293T cells, co-IP assay showed an interaction between ETHE1 and STAT3 (Fig. 4E). Furthermore, the interaction between endogenous ETHE1 and STAT3 was verified using anti-ETEH1 or anti-STAT3 antibodies in co-IP assays (Fig. 4F). In addition, in vitro Flag-pull down assay further elaborated that ETHE1 directly interacted with STAT3 (Fig. 4G).

Considering that phosphorylated STAT3 primarily localizes within the nucleus and initiates the transcription of downstream genes, we speculated whether ETHE1 could influence the nuclear STAT3 accumulation. Nuclear and cytoplasmic protein extraction assay was performed and the results delineated that ETHE1 overexpression notably decreased nuclear STAT3 protein levels (Fig. 4H). Meanwhile, downregulation of ETHE1 enhanced the nuclear translocation of STAT3 (Fig. 4I). Furthermore, cellular immunofluorescence analysis showed more STAT3 protein nuclear localization in ETHE1 knockdown SW48 cells compared to shNC SW48 cells (Fig. 4J). Taken together, our results reveal that ETHE1 interacts with STAT3 and impacts the activation of the STAT3 signaling pathway.

### ETHE1 downregulation-induced angiogenesis in CRC depends on STAT3/VEGF-A pathway

**Fig. 5:**
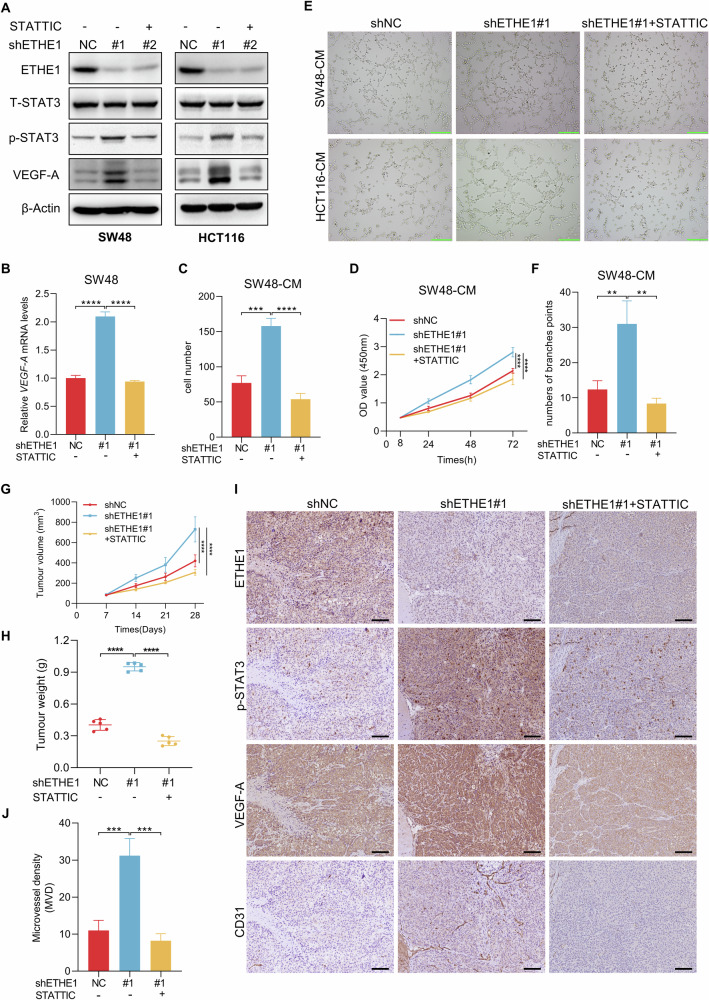
STAT3/VEGF-A pathway contributes to ETHE1 downregulation-induced tumor angiogenesis. **A** The protein level of VEGF-A, T-STAT3 and p-STAT3 was detected by IB assay in the indicated cells treated with or without STATTIC (10 μM, 48 h). **B** The mRNA level of *VEGF-A* detected by qRT-PCR assays in the indicated cells treated with or without STATTIC (10 μM, 48 h) (*n* = 3). **C** Quantitative analysis of Transwell cell migration assay in HUVECs treated with indicated CM (*n* = 3). **D** The viability of HUVECs treated with indicated CM was evaluated by using CCK8 assay (*n* = 3). **E** Representative images of tubule formation assay in HUVECs treated with indicated CM. Green scale bar, 100 μm. **F** Quantitative analysis of tubule formation assay in HUVECs treated with indicated CM (*n* = 3). **G** Tumor volumes were measured in xenografts generated from SW48 cells silenced with control (shNC) or ETHE1 shRNA#1, treated with either vesicle or STATTIC (3.75 mg/kg, every 2 days) via intratumoral injection (*n* = 5). **H** Tumor weights were measured in xenografts generated from SW48 cells silenced with control (shNC) or ETHE1 shRNA#1, treated with either vesicle or STATTIC (3.75 mg/kg, every 2 days) via intratumoral injection (*n* = 5). **I** IHC staining of xenograft tumors generated using anti-ETHE1, anti-p-STAT3, anti-VEGF-A, and anti-CD31 antibodies. Black scale bar, 50 μm. **J** Quantitative analysis of the Microvessel density of subcutaneous tumors according to the staining status of CD31 (*n* = 5). All immunoblots were performed three times, independently, with similar results. Data are represented as mean ± s.d. **p < 0.01, ***p < 0.001, ****p < 0.0001, by (**B**, **C**, **F**, **H**, **J**) one-way ANOVA with Tukey’s test and (**D**, **G**) two-way ANOVA with Tukey’s test.

Previous research has suggested that STAT3 can promote the transcription of VEGF-A to potentiate tumor angiogenesis [18]. To confirm the reliance of ETHE1 downregulation-mediated angiogenesis in CRC on STAT3/VEGF-A signaling, we selected STATTIC, a potent inhibitor of STAT3 signaling known to repress the phosphorylation levels of STAT3 [19]. Treatment of ETHE1-knockdown SW48 and HCT116 cells with STATTIC eliminated the increase in VEGF-A protein expression induced by ETHE1 silencing (Fig. 5A). Consistently, mRNA levels of *VEGF-A* showed the similar results (Fig. 5B, Supplementary Fig. 3A). Subsequently, we examined whether the effects of ETHE1 on migration, proliferation, and tube formation of HUVECs necessitated STAT3 activation. When STAT3 activation was blocked, the CM of ETHE1 knockdown cells failed to enhance migratory and proliferative abilities of HUVECs (Fig. 5C, D, Supplementary Fig. 3B–F). Moreover, HUVECs incubated with the CM from ETHE1 silenced SW48 and HCT116 cells exhibited more tube formation, which was reversed by inhibiting STAT3 activation (Fig. 5E, F, Supplementary Fig. 3G). Moreover, a xenograft model was used to confirm these effects in vivo. Tumors originating from ETHE1 knockdown SW48 cells exhibited a faster growth rate than those from shNC cells. However, this promotion effect of ETHE1 silencing was diminished by treatment with STATTIC (Fig. 5G). After the mice were sacrificed at the 28th day, all subcutaneous tumors were weighed, and tumor tissues were used in IHC assays. Heavier and larger tumors were observed in the ETHE1 knockdown groups, which was reversed by the treatment with STATTIC (Fig. 5H, Supplementary Fig. 3H). Moreover, IHC analysis revealed that STATTIC treatment reduced the expression of VEGF-A despite downregulating the expression of ETHE1 (Fig. 5I). The increase of CD31 staining and microvessel density were also eliminated by STATTIC treatment (Fig. 5I, J). These findings collectively indicate that the activation of the STAT3/VEGF-A pathway contributes to ETHE1 downregulation-induced angiogenesis both in vitro and in vivo.

### ETHE1 impedes STAT3 activation via stabilizing the interaction between TC45 and STAT3

**Fig. 6:**
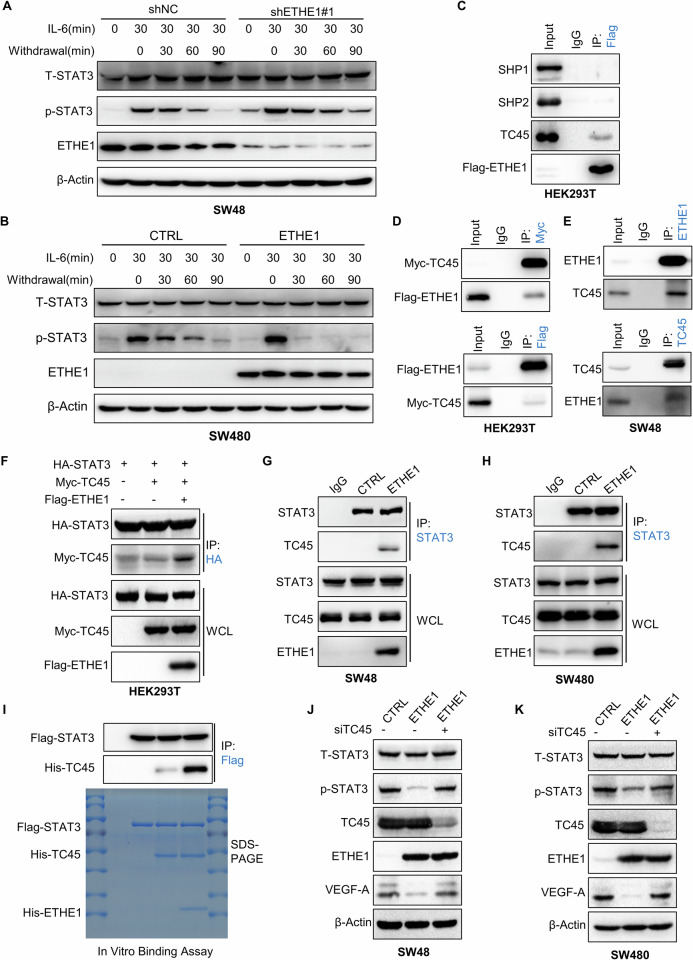
ETHE1 hinders STAT3 activation by enhancing the interaction of TC45-STAT3. **A**, **B** Indicated cells were treated with or without IL-6 (10 ng/mL) for 30 min, followed by various durations of starvation (indicated as withdrawal). WCL were collected for IB analysis. **C** HEK293T cells were transfected with Flag-ETHE1. WCL were collected for IP with anti-Flag beads, followed by IB analysis. **D** HEK293T cells were transfected with Myc-TC45 and Flag-ETHE1 plasmids, WCL were collected for IP with anti-Myc beads or anti-Flag beads, followed by IB analysis. **E** WCL of SW48 cells were collected for IP with anti-TC45 or anti-ETHE1 antibody, followed by IB analysis. **F** HEK293T cells were transfected with HA-STAT3 plasmid, then transfected with vector, Flag-ETHE1, or Myc-TC45 plasmids. WCL were collected for IP with anti-HA beads, followed by IB analysis. **G**, **H** WCL were collected from the SW48 and SW480 cells and conducted by IP assay using IgG or anti-STAT3 antibody, followed by IB analysis. **I** In vitro binding assay was performed. Purified Flag-STAT3 was incubated with TC45 in the absence or presence of purified His-ETHE1 and pulled down using anti-Flag beads, followed by IB analysis. **J**, **K** The protein level of VEGF-A, T-STAT3 and p-STAT3 detected by IB assay in the indicated cells treated with or without silencing TC45. All immunoblots were performed three times, independently, with similar results.

To differentiate whether ETHE1 inhibits STAT3 phosphorylation or promotes STAT3 dephosphorylation, we conducted cytokine stimulation-withdrawal experiments in cells with stably high or low expression of ETHE1. We found that in ETHE1 overexpressing cells, p-STAT3 protein level was comparable to the initial level after 30 min of IL-6 withdrawal. However, in ETHE1-low expression cells, the phosphorylation level of STAT3 remained higher than that of shNC cells after 90 min of IL-6 withdrawal (Fig. 6A, B). These results indicated that ETHE1 accelerated STAT3 dephosphorylation. Subsequently, we aimed to elucidate the precise molecular mechanisms underlying ETHE1-mediated STAT3 dephosphorylation. Given that ETHE1 lacks diphosphatase activity and is known as a nuclear-cytoplasmic shuttle protein, we hypothesized that ETHE1 could affect the dephosphorylation of STAT3 by facilitating its binding with relevant phosphatases. It has been reported that SHP1, SHP2, and TC45 are capable of mediating the dephosphorylation of STAT3. Consequently, we conducted experiments to ascertain the potential interaction between ETHE1 and these proteins. Our findings revealed that ETHE1 could bind to TC45, while no interaction was observed with SHP1 or SHP2 (Fig. 6C). Flag-ETHE1 and Myc-TC45 plasmids were transfected into HEK293T cells and an interaction between ETHE1 and TC45 was detected by Co-IP assays (Fig. 6D). In addition, the association between endogenous ETHE1 and TC45 was confirmed in SW48 cells by Co-IP with anti-ETHE1 or anti-TC45 antibody (Fig. 6E).

Given the results that ETHE1 could directly interact with STAT3 (Fig. 4F), we speculated whether ETHE1 could form a complex with STAT3 and TC45 and then stabilize the interaction between them. We transfected HA-STAT3 and MYC-TC45 plasmids into HEK293T cells, followed by transfection with either empty vector or Flag-ETHE1 plasmid, and performed Co-IP assays using anti-HA magnetic beads. The results showed enhanced interaction of STAT3 and TC45 in the presence of Flag-ETHE1 (Fig. 6F). Meanwhile, we found that endogenous ETHE1 protein could facilitate the binding of TC45 and STAT3 in SW48 and SW480 cells (Fig. 6G, H). The direct effect of ETHE1 on the interaction between STAT3 and TC45 was further verified by in vitro binding assays, and the results revealed a strong interaction between STAT3 and TC45 in the presence of ETHE1 protein (Fig. 6I). We further investigated whether the effect of ETHE1 on VEGF-A expression depended on the STAT3/TC45 signaling pathway. By using siRNA to silence TC45 in ETHE1 overexpressing cells, we found that the decrease in VEGF-A caused by overexpressing ETHE1 was completely reversed (Fig. 6J, K). Taken together, these results suggest that ETHE1 regulates the dephosphorylation of STAT3 by promoting the binding of STAT3 and TC45, and the inhibition of VEGF-A expression by ETHE1 is contingent on the STAT3/TC45 signaling pathway.

### Correlation between the expression of ETHE1 and VEGF-A and CRC patients’ overall survival

**Fig. 7:**
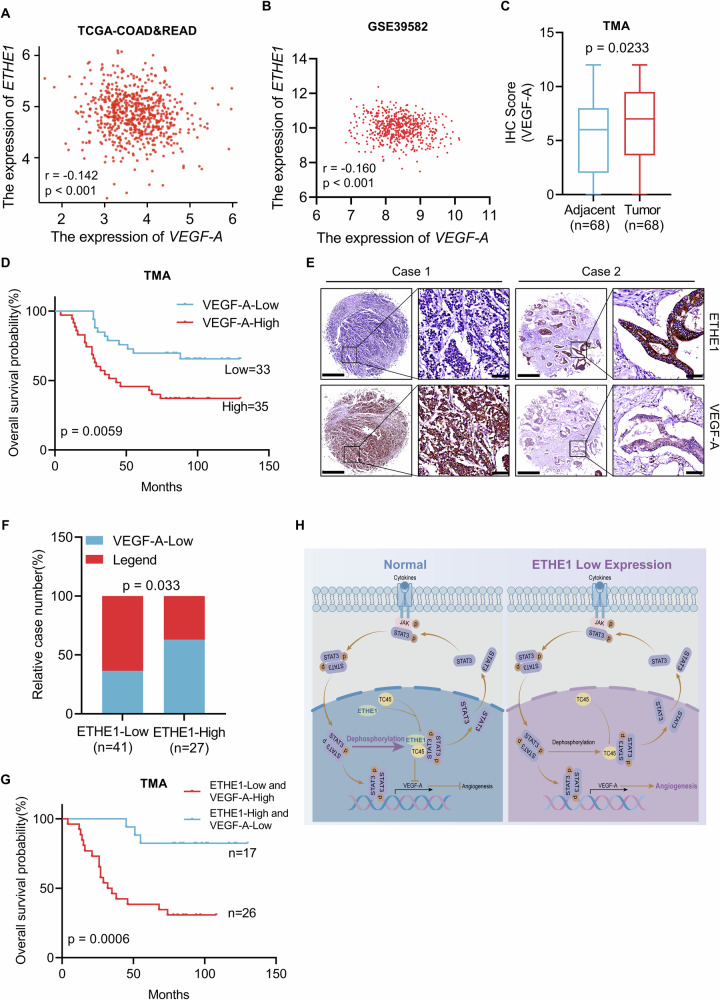
Correlation between ETHE1 and VEGF-A expression and association with CRC patients’ overall survival. **A**, **B** The correlation between the mRNA expression of *ETHE1* and *VEGF-A* was determined by the Pearson correlation coefficient test in in the TCGA COAD and READ datasets and the GSE39582 dataset, *p* < 0.001, *r* = −0.142 and *p* < 0.001, *r* = −0.160. **C** Quantitative analysis of The IHC score of ETHE1 in the CRC TMA. Student’s two-tailed t test, *p* = 0.0233. **D** Kaplan–Meier Overall survival (OS) analysis in the according to VEGF-A expression in the CRC tissues microarray. Log-rank test, *p* = 0.0059. **E** Representative images of ETHE1 and VEGF-A expression from the CRC TMA. Overview image with black scale bar: 500 μm and enlarged image with black scale bar: 100 μm. **F** Quantitative IHC staining score showing the correlation between ETHE1 and VEGF-A using the TMA. Chi-square test, *p* = 0.033. **G** Kaplan–Meier Overall survival (OS) analysis in the according to ETHE1 and VEGF-A expression in the TMA. Log-rank test, *p* = 0.0006. **H** The working model proposes that ETHE1 dampens colorectal cancer angiogenesis by promoting TC45 Dephosphorylation of STAT3 to inhibit VEGF-A expression.

We assessed the mRNA expression of *ETHE1* and *VEGF-A* in the TCGA database and GEO datasets (GSE39582) and found a significant negative correlation of *ETHE1* and *VEGF-A* (Fig. 7A, B). Furthermore, we detected the expression of VEGF-A in CRC tissue microarray by IHC assays. Consistent with previous study [20, 21], VEGF-A was highly expressed in CRC patient’s tumor tissue compared with adjacent tissues and Kaplan–Meier survival analysis revealed that high VEGF-A expression was associated with a significantly longer overall survival time (Fig. 7C, D). In addition, our results demonstrated that the protein levels of VEGF-A were negatively correlated with ETHE1 expression (Fig. 7E, F). To further reveal the clinical significance of our study, we conducted a prognostic analysis comparing patients with low ETHE1 levels and high VEGF-A levels to those with high ETHE1 levels and low VEGF-A levels. The results indicated that individuals with low ETHE1 and high VEGF-A expression exhibited a worse prognosis compared to those with high ETHE1 and low VEGF-A levels. (Fig. 7G). Overall, these data suggest that ETHE1 negatively correlates with VEGF-A expression in CRC patients and the combination of low ETHE1 and high VEGF-A expression can serve as a more effective prognostic indicator for CRC patients.

## Discussion

ETHE1 has been extensively explored in ethylmalonic encephalopathy, but its investigation in CRC is limited [12, 22]. In our study, we identified ETHE1 as a potential suppressor of CRC using the publicly available data, clinically fresh paired samples, and paired CRC tissue microarray. Moreover, we found ETHE1 expression could serve as an independent and meaningful prognostic factor for patients with CRC. Consistent with our findings, a distinct meta-analysis revealed that downregulation of ETHE1 in colonic adenocarcinoma could enhance energy production under hypoxia conditions [23]. Conversely, Ozluk E et al reported an increase in ETHE1 expression in colon cancer compared to benign colonic epithelium [24]. This disparity may stem from the use of different clinical samples. While Ozluk E’s study utilized unpaired clinical samples, with a considerably smaller number of normal samples in comparison to tumor samples, our research employed paired samples, enabling a more accurate assessment of ETHE1 expression in CRC.

ETHE1 was initially identified to facilitate hydrogen sulfide catabolism. In addition, the dysfunctional ETHE1 has an impact on redox regulation in human fibroblasts, as well as lipid catabolism and cytoskeleton dynamics in mice tissue [25, 26]. In our current study, we uncovered a distinct biological function of ETHE1 that was independent of its enzymatic activity in CRC. Our data demonstrated that the downregulation of ETHE1 played an important role in tumor growth and tumor angiogenesis in vitro and in vivo. Moreover, there exists a negative correlation between ETHE1 expression and VEGF-A levels in CRC patients. Individuals with low ETHE1 and high VEGF-A expression exhibited a worse prognosis compared to those with high ETHE1 and low VEGF-A levels. These data suggest ETHE1 has the potential to serve as a biomarker for anti-VEGF targeted angiogenesis therapy in CRC. Additionally, beyond its role in promoting angiogenesis, further comprehensive research into other potential biological functions of ETHE1 is needed.

Transcription factors play a critical role in regulating the expression of VEGF-A, with STAT3 known for its capacity to activate VEGF-A in cancer cells [18, 27]. Moreover, STAT3 activation governs a variety of cellular processes, including cell proliferation, migration, and angiogenesis [28]. Thus, we detected whether ETHE1 could influence the activation of STAT3 by its inhibition or overexpression. Our data revealed that downregulation of ETHE1 significantly increased phosphorylation of STAT3, while overexpression of ETHE1 markedly decreased STAT3 phosphorylation and nuclear localization in CRC cells. Furthermore, inhibiting STAT3 phosphorylation completely reversed the impact of ETHE1 knockdown on VEGF-A expression and angiogenesis in vivo and in vitro, consistent with a previous finding of VEGF-independent angiogenesis via STAT3 signaling [29]. These results suggest ETHE1 downregulation-induced angiogenesis in CRC depends on STAT3/VEGF-A pathway.

Mounting evidence suggests STAT3 signaling is negatively regulated by protein tyrosine phosphatases (PTPs), including SHP1, SHP2, and TC45 [30]. SHP1 and SHP2 indirectly inhibit STAT3 activity in the cytoplasm by dephosphorylating protein tyrosine kinase JAK, whereas TC45 directly dephosphorylates STAT3 in the nucleus [31–33]. Recent researches indicate that the STAT3/TC45 pathway in various tumor biological functions such as proliferation, metastasis, and radio-resistance [34–37]. Specifically, TRIM59 mediated EGFR stimulation of STAT3 by inhibiting TC45 dephosphorylation of STAT3 in the nucleus, thereby maintaining transcriptional activation of STAT3 and promoting tumorigenesis [34]. Additionally, it has been observed that radiotherapy enhances the interaction between TRIM32 and STAT3, which suppressed TC45-induced dephosphorylation of STAT3, resulting in increased STAT3 transcriptional activation and radio-resistance in TNBC [37]. Our results identify a novel signaling network, in which ETHE1 inhibits oncogenic signaling by promoting the formation of the STAT3-TC45 complex, thereby restraining STAT3 phosphorylation and activation.

In conclusion, our study initially demonstrates the crucial involvement of ETHE1 in tumor angiogenesis and uncovers a previously unrecognized mechanism whereby ETHE1 facilitates TC45-mediated dephosphorylation of STAT3, thereby suppressing the oncogenic effects of STAT3 signaling in CRC. The newly established roles of ETHE1 in tumor angiogenesis provide a rationale for ETHE1 as a novel prognostic indicator for CRC patients and a potential target for further therapeutic investigation. Nevertheless, our study concentrates on elucidating the downstream pathway of ETHE1 while neglecting to address the regulation of ETHE1 in CRC. The reason for the downregulation of ETHE1 in CRC needs to be further explored.

## Materials and methods

### Tissue samples and Immunohistochemistry

The present study obtained approval from the Ethics Committee of Tongji Hospital (TJ‐IRB20220723). Paired CRC tissues for this study were obtained from the Wuhan Tongji Hospital. The patient characteristics of tissue microarrays were summarized in Table 1. Tissues from subcutaneous tumors and tissue microarrays were used to conduct the immunohistochemistry (IHC). The sections were incubated with the indicated primary antibodies overnight at 4 °C and then with the second antibodies. In tissue microarrays, the intensity score was classified as: 0 (negative), 1 (weak), 2 (moderate), and 3 (strong), and the density score was indicated as: 0–5% = 1, 6–30% = 2, 31–70% = 3, and 71–100% = 4. Then, the total IHC scores were generated by multiplying these two scores (0 to 12). The score from 0 to 6 was regarded as low expression, while the score more than 6 was assigned to a high expression. All cases were scored by two independent pathologists.

### Cell lines and cell culture

Human embryonic kidney cells HEK293T, human CRC cell lines (SW48, SW480, and HCT116), and human umbilic vein endothelial cells (HUVEC) were purchased from American Type Culture Collection. These cells grew in Dulbecco’s Modified Eagle Medium (DMEM), Leibovitz’s L-15 medium, and RPMI-1640 Medium with 10% FBS, 1% penicillin and 1% streptomycin. All cells were cultured in a humidified cell culture incubator containing 5% CO_2_ at 37 °C.

### Reagents

STATTIC (HY-13818), IL-6 (HY-P7044), Anti-HA Magnetic Beads (HY-K0201), Anti-Flag Magnetic Beads (HY-K0207), Anti-c-Myc Magnetic Beads (HY-K0206) and Magnetic Beads Protein A/G Magnetic Beads (HY-K0202) were obtained from MedChemExpress (MCE). Matrigel Matrix (354230) was purchased from Corning. Antibodies against ETHE1 (A10142; Abclonal, sc-393869; Santa Cruz), STAT3 (9139; Cell Signaling Technology), p-STAT3 (4113S; Cell Signaling Technology), VEGF-A (19003-1-AP; Proteintech), TC45 (A1808; Abclonal), β-Actin (AC038; Abclonal), Flag tag (14793S; Cell Signaling Technology), HA tag (51064-2-AP; Proteintech), Myc tag (AE010; Abclonal) and Lamin B1 (A16909; Abclonal) were commercially purchased.

### Plasmids

The genes of *ETHE1*, *STAT3*, and *TC45* were inserted into pcDNA3.1(+) vector respectively. The lentiviral plasmids pLVX-ETHE1-puro was constructed by cloning *ETHE1* genes into the pLVX-puro vector. The lentiviral plasmids pLKO.1-ETHE1-puro was constructed by inserting the *ETHE1* shRNA into the pLKO.1-puro vector. The short hairpin RNA (shRNA) sequences targeting human *ETHE1* are: 5ʹ- CAGCAGATAGACTTTGCTGTT-3ʹ, 5ʹ- TGCTCACGATTACCATGGGTT-3ʹ.

### Construction of lentiviral-infected cell lines

After co-transfecting HEK293T cells with pLKO.1-ETHE1-puro, PMD2.G, and psPAX2 for 72 h, we collected the cell culture supernatant to infect the indicated tumor cells for 2 days. Next, the cells were treated with 2.0 μg/ml puromycin for 48 h and verified by Western blot and qPCR. Likewise, cell lines with stable overexpression of ETHE1 were constructed using the same method.

### Western Blot, Immunoprecipitation, and Immunofluorescence analysis

For western blot assay, total proteins were isolated from the indicated tissues or cells by NP40 buffer containing protease inhibitors and phosphatase inhibitors. After quantification and denaturation of the protein, equal amounts of protein were subjected to gel electrophoresis and transfer. Next, the membranes were blocked by 5% skim milk for 1 h, followed by incubated with specific antibodies overnight at 4°C. After incubated at second antibodies for 1 h, the membranes were detected by ECL. For immunoprecipitation (IP), the indicated lysates were mixed with Anti-Flag, Anti-HA beads, Anti-c-Myc beads or Protein A/G beads conjugated with specific antibodies and rotated overnight at 4 °C. Beads were washed five times with lysis buffer and followed by western blot assay. For immunofluorescence (IF) assays, the indicated cells were cultured on coverslips and fixed with 4% paraformaldehyde for 20 min. After permeabilized with 0.3% Triton X-100, the samples were blocked with 2% BSA, and then stained with specific antibodies overnight at 4 °C. Subsequently, the samples were incubated with corresponding fluorescently labeled secondary antibodies (Dylight 549, Goat Anti-Mouse IgG) for 2 h at room temperature, and followed by staining with 4,6-diamidino-2-phenylindole (DAPI). The images were obtained by a confocal fluorescence microscope (Olympus).

### In vitro binding assays

Flag-STAT3 protein produced using Baculovirus expression system was purchased from Active Motif (81095), His-ETHE1 was obtained from SinoBiological (14681-H07E), and recombinant TC45 protein with His tag was purified from *E. coli*. In vitro binding assays, Flag-STAT3 protein was incubated with His-ETHE1 protein alone or with His-ETHE1 protein and His-TC45 protein together and then rotated with Anti-Flag Magnetic Beads overnight at 4°C. Beads were washed five times with lysis buffer and followed by western blot assay.

### Quantitative real-time PCR

The detailed procedures were performed as previously described. The primers used were as follows: 5ʹ- GCAGTGGGTATTCACAGCATAG-3ʹ (*ETHE1*-forward), 5ʹ- GCAGTGGGTATTCACAGCATAG-3ʹ (*ETHE1*-reverse), 5ʹ-AGGGCAGAATCATC ACGAAGT-3ʹ (*VEGF-A*-forward), 5ʹ- AGGGTCTCGATTGGATGGCA-3ʹ (*VEGF-A*-reverse), 5ʹ-GAAGAGTTGGATACTCAGCGTC-3ʹ (*TC45*-forward), 5ʹ-TGCAGTT TAACACGACTGTGAT-3ʹ (*TC45*-reverse), 5ʹ-ACAACTTTGGTATCGTGGAAGG-3ʹ (*GAPDH*- forward), 5ʹ-GCCATCACGCCACAGTTTC-3ʹ (*GAPDH*- reverse).

### Conditioned medium (CM) preparation

When the indicated CRC cell lines reached approximately 80% confluence, the medium was replaced with fresh medium and incubated for 24 h. Subsequently, the media were collected, centrifuged at 240 × g at 4 °C for 5 min to remove cellular debris, and stored at −80 °C. This conditioned medium (CM) was utilized for subsequent assays related to HUVECs.

### The scratch assay

HUVECs were seeded into 6-well plates. Upon reaching approximately 90% confluence, a wound was generated by scratching the cell layer using a sterile 10 μL pipette tip, followed by exposure to conditioned medium (CM) from CRC cells. Migration ability was assessed by measuring the width of the gap between the wound edges under a microscope at 0 h and 12 h.

### HUVEC proliferation, migration assays, and tubule formation

HUVEC proliferation, migration assays, and tubule formation were performed as described [38]. For proliferation assay, we performed CCK8 assay to detect the proliferative capacity of HUVEC. Briefly, 3 × 10^3^ cells were seeded in 96-well plates with the indicated CM treatment. After incubated with CCK8 solution for 1 h, we measured the absorbance at the indicated time. For migration assay, about 5 × 10^4^ cells were resuspended in serum-free medium and placed in the upper chamber, and then 700 μl of indicated CM was added to the lower chamber. After incubation for 16 h, cells were stained with crystal violet and observed under a microscope. For tubule formation assay, Matrigel matrix was added 50 μl/well in a 96-well plate and incubate at 37 °C for 30 min. 4 × 10^4^ cells were suspended in 100 μl CM and added onto the solidified Matrigel matrix. After 6 h of incubation, the cells were observed and photographed under a microscope.

### In vivo Matrigel plug assay

After protein quantification, mix the equal amount of CM and Matrigel together. About 700 μl Matrigel mixture was subcutaneously injected into Balb/c nude mouse. One week later, the Matrigel plugs were extracted and measured the hemoglobin concentration.

### Mouse tumor xenograft model

Animal studies were reviewed and approved by the Committee on the Ethics of Animal Experiments of Tongji Hospital (TJH-201904007). BALB/c nude mice were purchased from GemPharmatech Co., Ltd (Jiangsu, China) and bred in an SPF-grade experimental animal room. The indicated cells were counted and suspended in the PBS, followed by mixed with Matrigel in a 1:1 ratio. Each 6-weeks-old BALB/c nude mouse was randomly divided into indicated groups (*n* = 5) and subcu0taneously injected with 100 μl cell suspension containing 5 × 10^5^ CRC cells. In some trials, vehicle or STATTIC was intratumorally injected every 2 days. After 28 days, the mice were sacrificed and xenografts were collected in a single blind procedure for follow-up tests.

### Statistical analysis

All statistical analyzes were analyzed using GraphPad 8.0. The two-sided unpaired Student’s t test, One-way ANOVA with Tukey’s test, or Two-way ANOVA with Tukey’s test was used to determine differences between groups. The chi-square test and the spearman test were performed where indicated. The Kaplan–Meier method and log-rank test were utilized to make a survival curve. *P* < 0.05 indicated the difference was statistically significant.

### Supplementary information

Supplementary Figure and legend

Original Data File

## Data Availability

The datasets used in the current study are available from the corresponding author on reasonable request.
