# Supplementary material for: ETHE1 dampens colorectal cancer angiogenesis by promoting TC45 Dephosphorylation of STAT3 to inhibit VEGF-A expression
Source: Cell Death Dis. 2024 Aug 28;15(8):631. doi: 10.1038/s41419-024-07021-w (PMC11358511; doi:10.1038/s41419-024-07021-w)
Supplement: Supplementary file 1 — Supplementary Figure and legend [file 41419_2024_7021_MOESM1_ESM.pdf]

ETHE1 dampens colorectal cancer angiogenesis by promoting TC45 Dephosphorylation of STAT3 to inhibit VEGF-A expression

Xiaowei She[1], Jialu Xu[2], Haokun Zhang[1], Chengxin Yu[1], Zejun Rao[1], Jiakun Zhang[1], Wenli Zhan[1], Fuqing Hu[1], Da Song[1], Haijie Li[1], Xuelai Luo[1], Guihua Wang[1,3], Junbo Hu[1], Senyan Lai[1]

1, GI Cancer Research Institute, Tongji Hospital, Huazhong University of Science and Technology, Wuhan, China.

2, Department of Endocrinology, Tongji Hospital, Tongji Medical College, Huazhong University of Science and Technology, Wuhan, China.

3, Tongji Medical College and State Key Laboratory for Diagnosis and Treatment of Severe Zoonotic Infectious Disease, Huazhong University of Science and Technology, Wuhan, 430030, Hubei, China.

To whom all correspondence should be sent:

Senyan Lai, GI Cancer Research Institute, Tongji Hospital, Huazhong University of Science and Technology, Wuhan, China. E-mail: laisenyan@hust.edu.cn

Key Words: ETHE1; VEGF-A; Angiogenesis; TC45/STAT3 pathway.

Running title: ETHE1 inhibits tumor angiogenesis through STAT3/TC45 pathway.

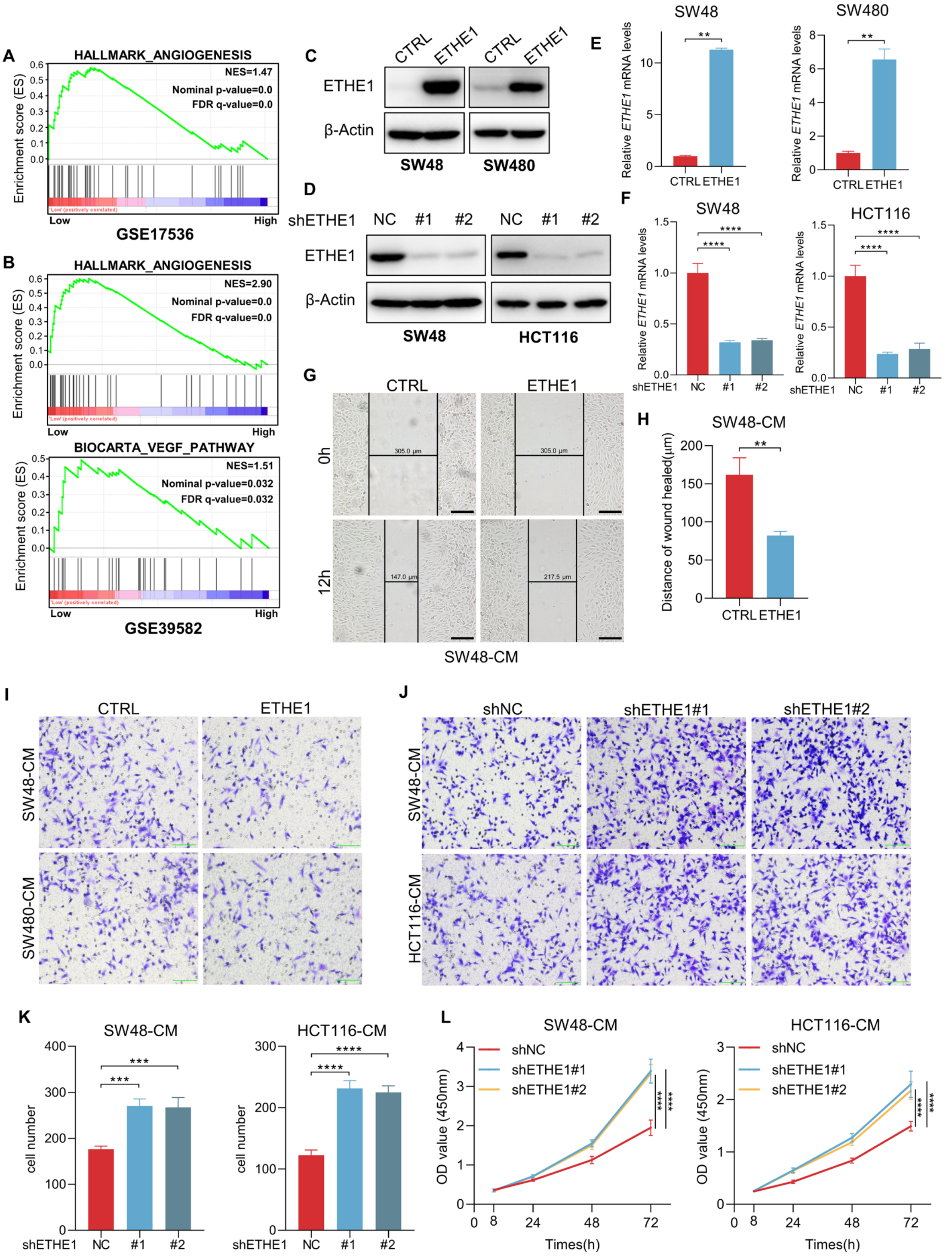

Supplementary Figure 1. ETHE1 inhibits tumor angiogenesis in vitro. A GSEA enrichment plots demonstrate the correlation between the expression of *ETHE1* and Angiogenesis in the GSE17536 datasets. **B** GSEA enrichment plots demonstrate the correlation between the expression of *ETHE1* and Angiogenesis (Up) or VEGF pathway (Down) in the GSE39582 datasets. **C** The protein level of ETHE1 detected

by IB assay in control or ETHE1 stable overexpression SW48 and SW480 cells. **D** The protein level of ETHE1 detected by IB assay in SW48 and HCT116 cells silenced with control (shNC) or ETHE1 shRNA (#1 and #2). **E, F** The mRNA level of *ETHE1* detected by qRT-PCR assays in control or ETHE1 stable overexpression SW48 and SW480 cells and SW48 and HCT116 cells silenced with control (shNC) or ETHE1 shRNA (#1 and #2) (n = 3). **G** Representative image of scratch assay in HUVEC cells treated with the indicated CM. Black scale bar, 100 μm. **H** Quantitative analysis of scratch assay from (G) at 24 h. **I, J** Representative images of Transwell cell migration assay in HUVECs treated with indicated CM. Green scale bar, 100 μm. **K** Quantitative analysis of Transwell cell migration assay from (I) and (E) (n = 3). **L** The viability of HUVECs treated with indicated CM evaluated by using CCK-8 assay (n = 3). All immunoblots were performed three times, independently, with similar results. Data are represented as mean ± s.d. $^{**}p < 0.01$, $^{***}p < 0.001$, $^{****}p < 0.0001$, by E, H) Student’s two-tailed t-test and F, K) one-way ANOVA with Tukey’s test and L) two-way ANOVA with Tukey’s test.

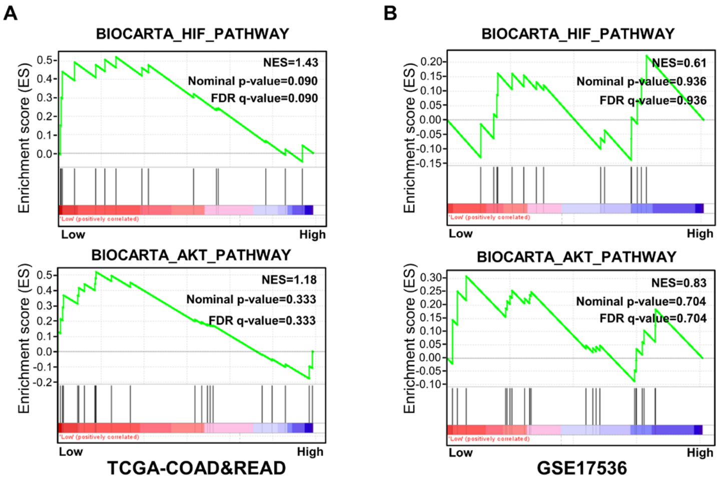

Supplementary Figure 2. ETHE1 has no correlation with HIF pathway and AKT pathway. **A** GSEA enrichment plots demonstrate the correlation between the expression of *ETHE1* and HIF pathway (Up) or AKT pathway (Down) in the TCGA COAD and READ datasets. **B** GSEA enrichment plots demonstrate the correlation between the expression of *ETHE1* and HIF pathway (Up) or AKT pathway (Down) in the GSE17536 datasets.

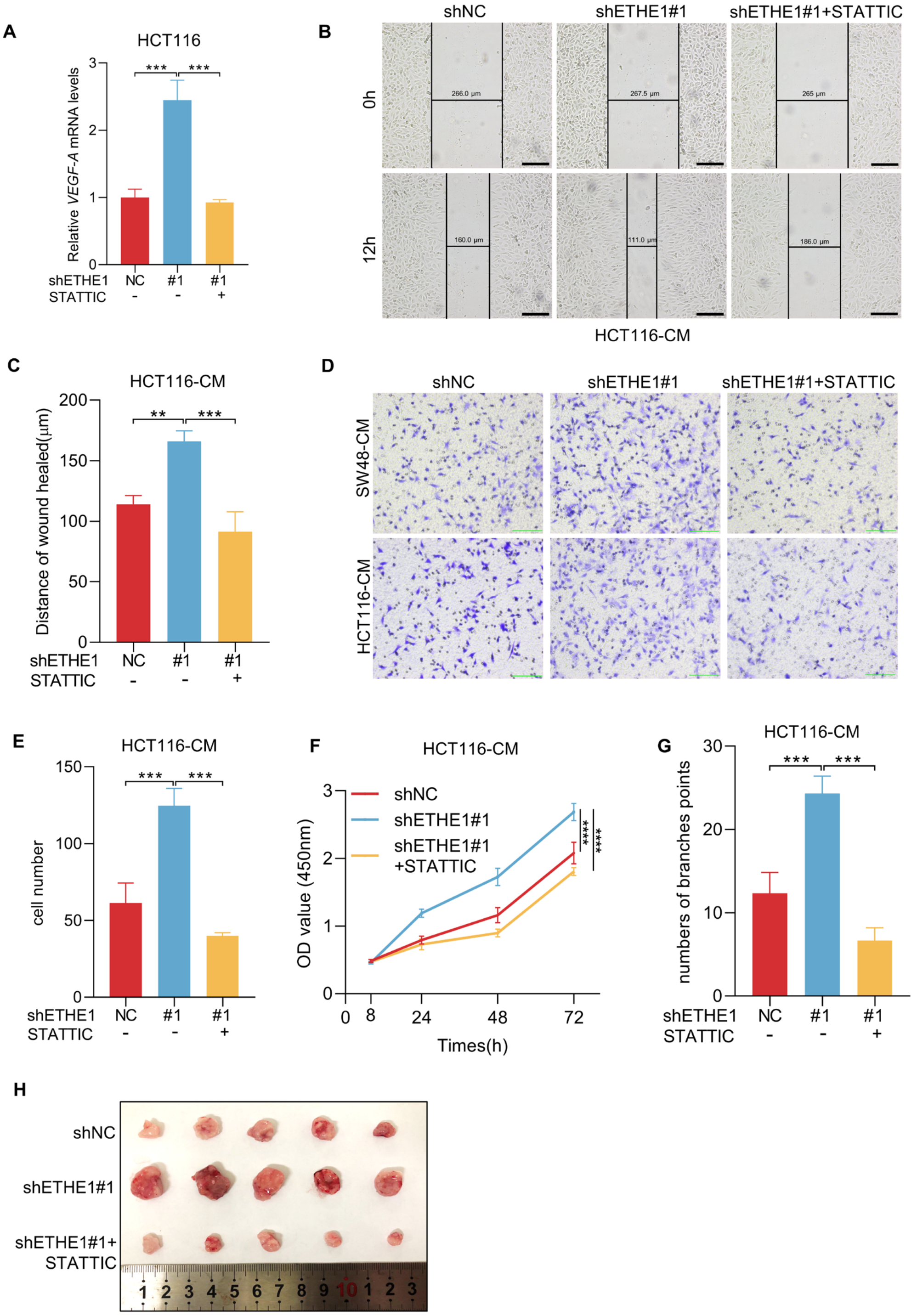

Supplementary Figure 3. STAT3/VEGF-A pathway contributes to ETHE1 downregulation-induced angiogenesis in CRC. A The mRNA level of *VEGF-A* detected by qRT-PCR assays in the indicated cells treated with or without STATTIC (10 μM, 48 h) (n = 3). **B** Representative image of scratch assay in HUVEC cells treated with the indicated CM. Black scale bar, 100 μm. **C** Quantitative analysis of

scratch assay from (B) at 24 h. **D** Representative images of Transwell cell migration in HUVECs treated with indicated CM. **E** Quantitative analysis of Transwell cell migration assay in HUVECs from (D) (n = 3). Green scale bar, 100 μm. **F** The viability of HUVECs treated with indicated CM evaluated by using CCK-8 assay (n = 3). **G** Quantitative analysis of tubule formation assay in HUVECs treated with indicated CM (n = 3). **H** Representative image of xenografts generated from SW48 cells silenced with control (shNC) or ETHE1 shRNA#1, treated with either vesicle or STATTIC (3.75 mg/kg, every 2 days) via intratumoral injection. Data are represented as mean ± s.d. ***p <0.001, ****p < 0.0001, by A, C, E, G) one-way ANOVA with Tukey's test and F) two-way ANOVA with Tukey's test.
