# Supplementary material for: ETHE1 dampens colorectal cancer angiogenesis by promoting TC45 Dephosphorylation of STAT3 to inhibit VEGF-A expression
Source: Cell Death Dis. 2024 Aug 28;15(8):631. doi: 10.1038/s41419-024-07021-w (PMC11358511; doi:10.1038/s41419-024-07021-w)

Figure.1D

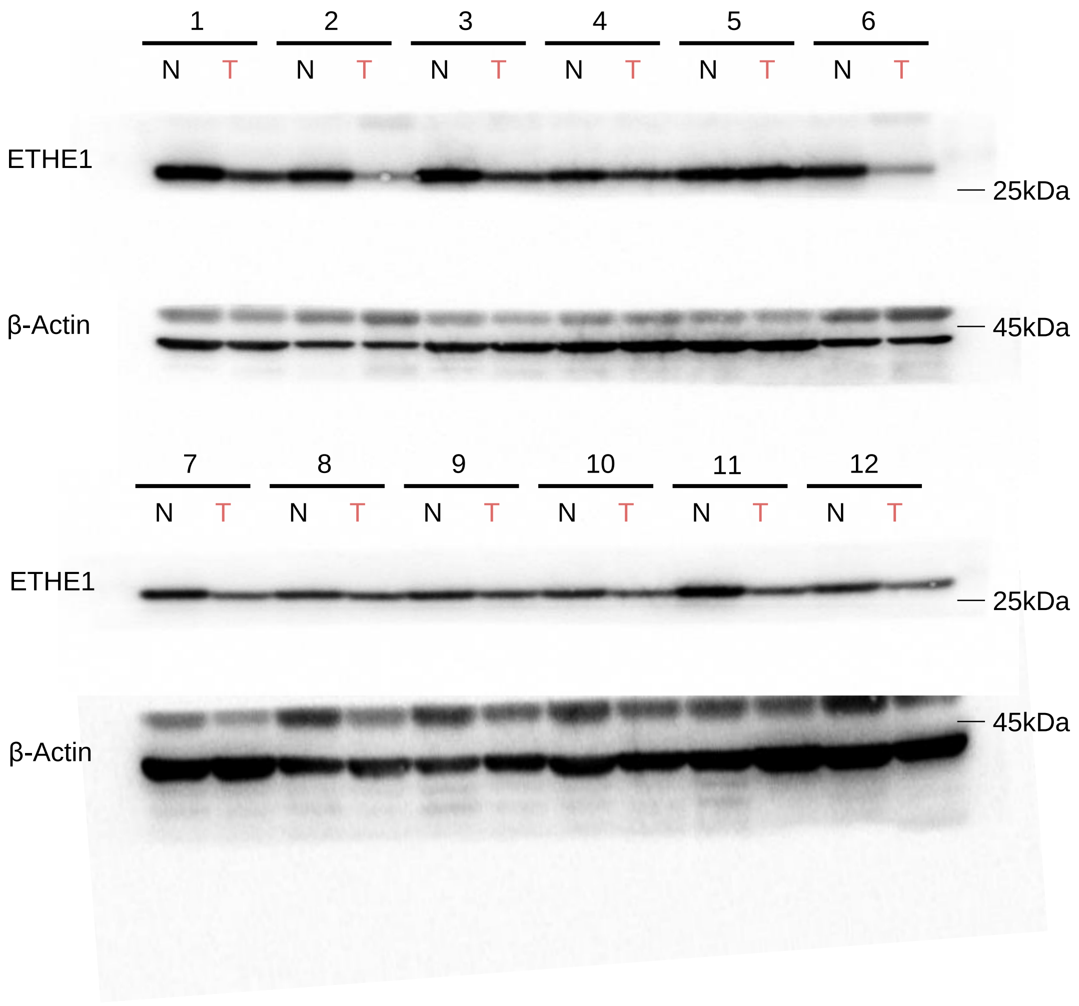
1
2
3
4
5
6
N T N T N T N T N T N T
ETHE1
25kDa
β-Actin
45kDa
7
8
9
10
11
12
N T N T N T N T N T N T
ETHE1
25kDa
β-Actin
45kDa

Figure.2B

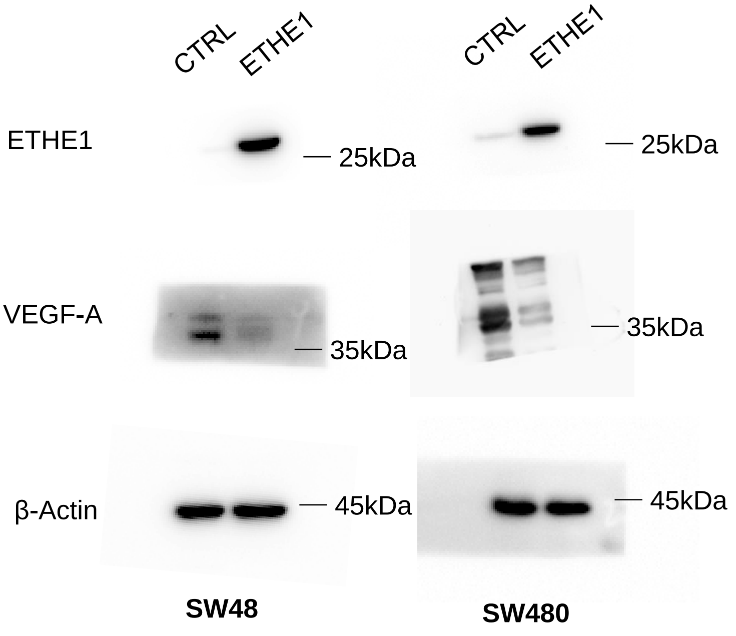

Figure.2C

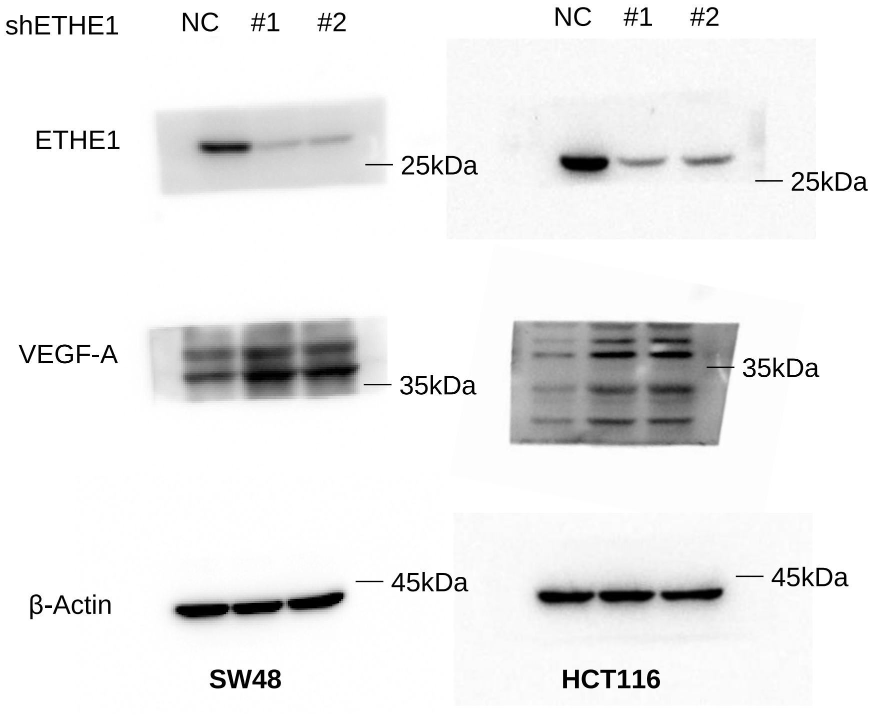

Figure.4B

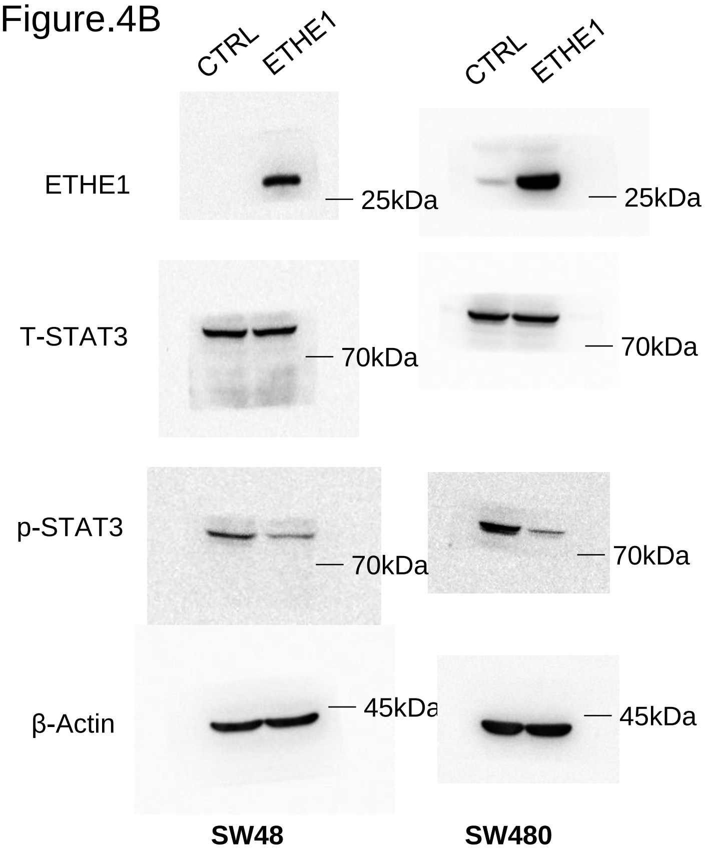

Figure.4C

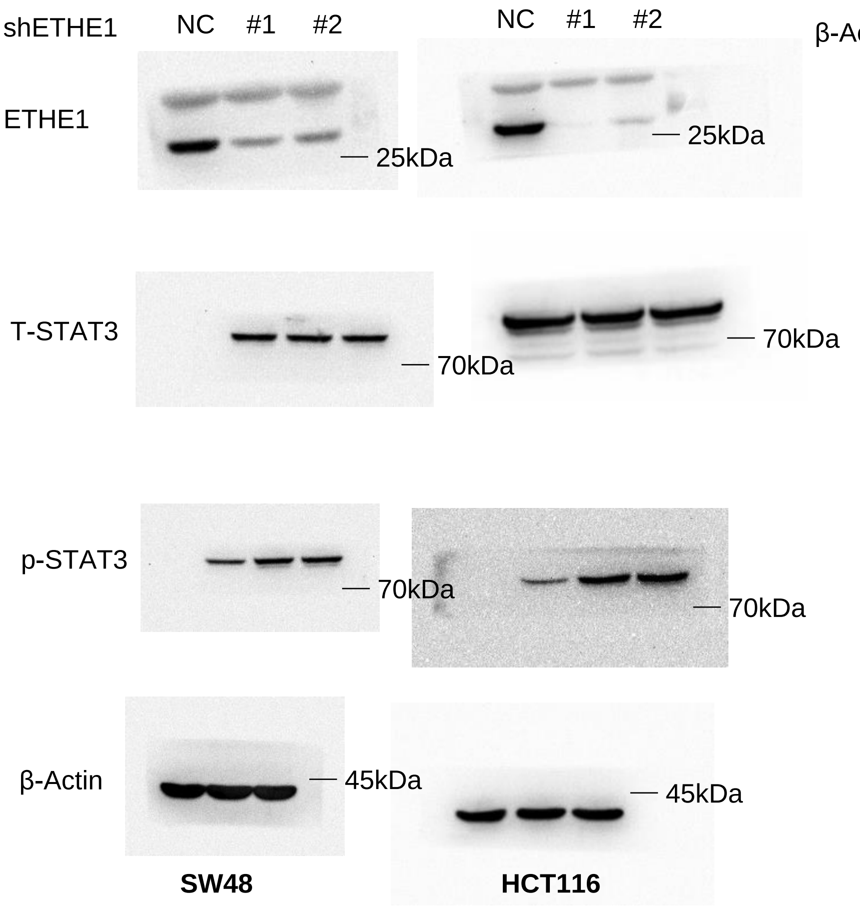

Figure.4D

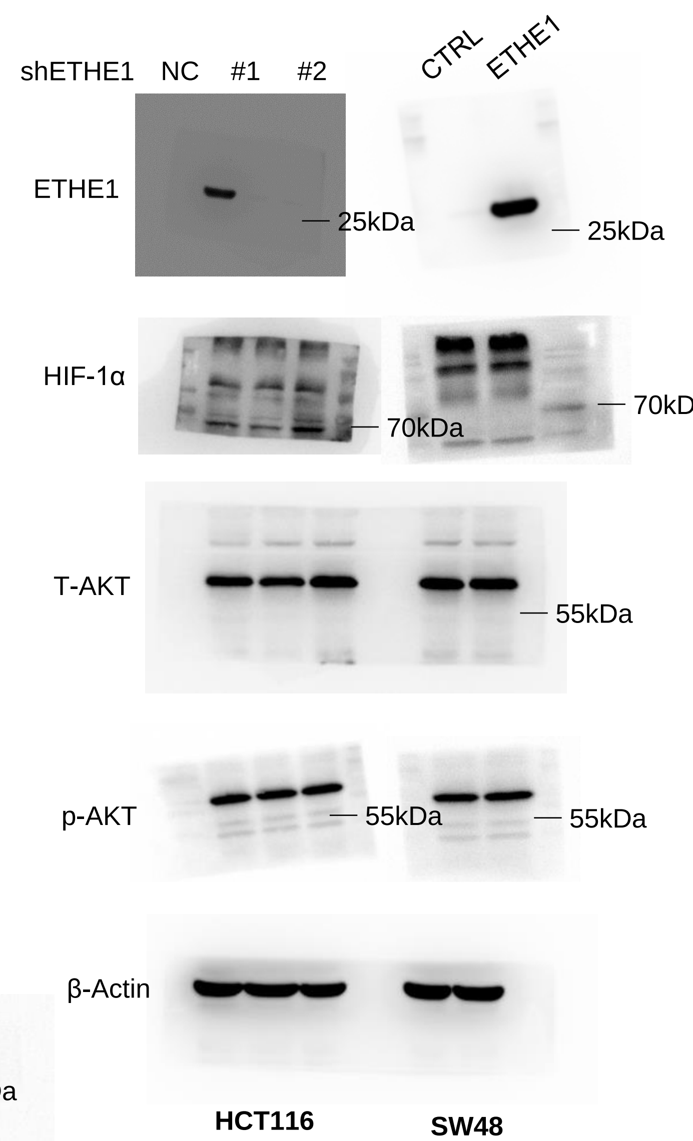

Figure.4E

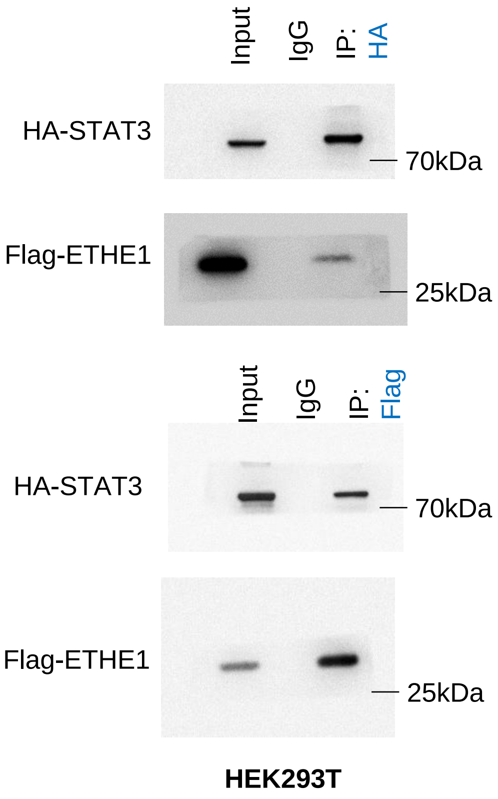

Figure.4G

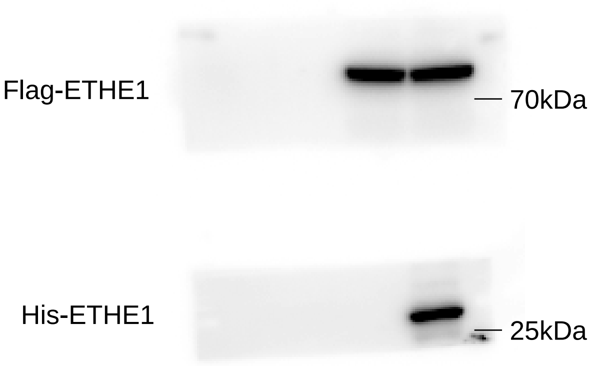

Figure.4F

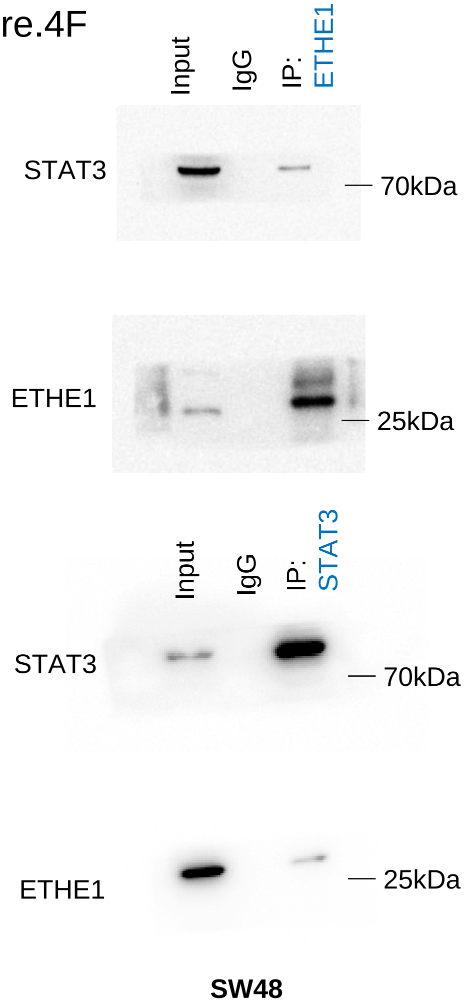

Figure.4H

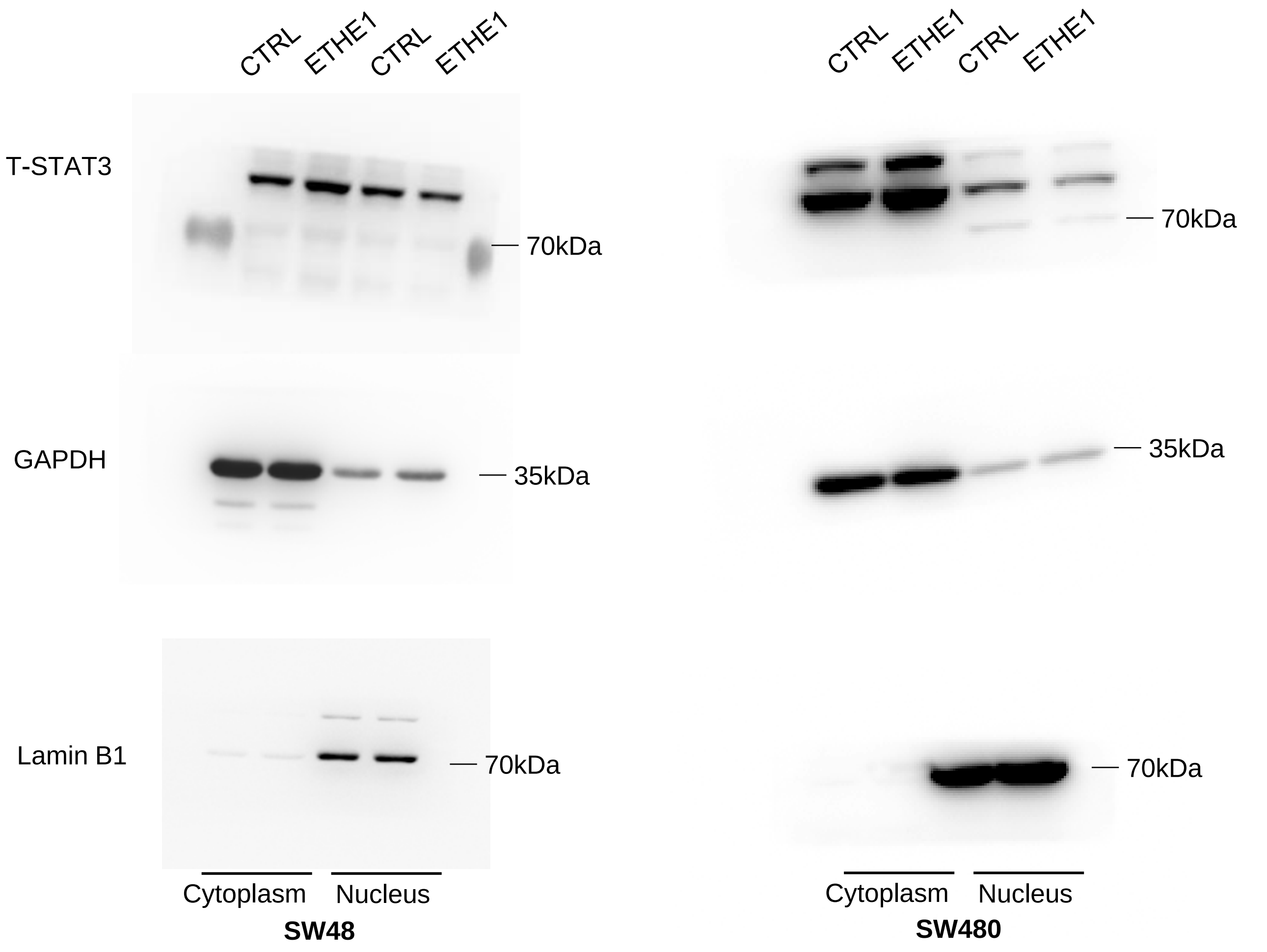

Figure.4I

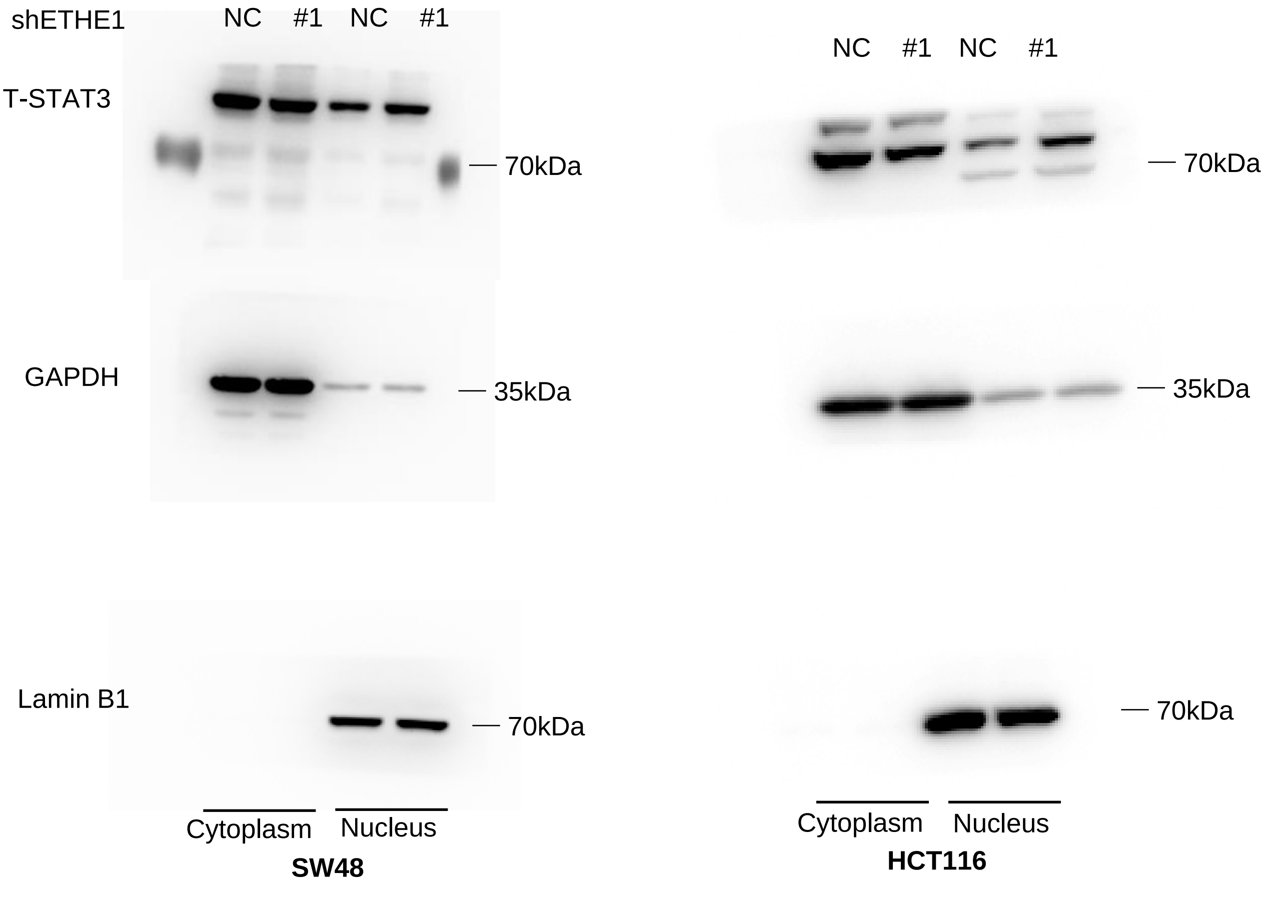

Figure.5A

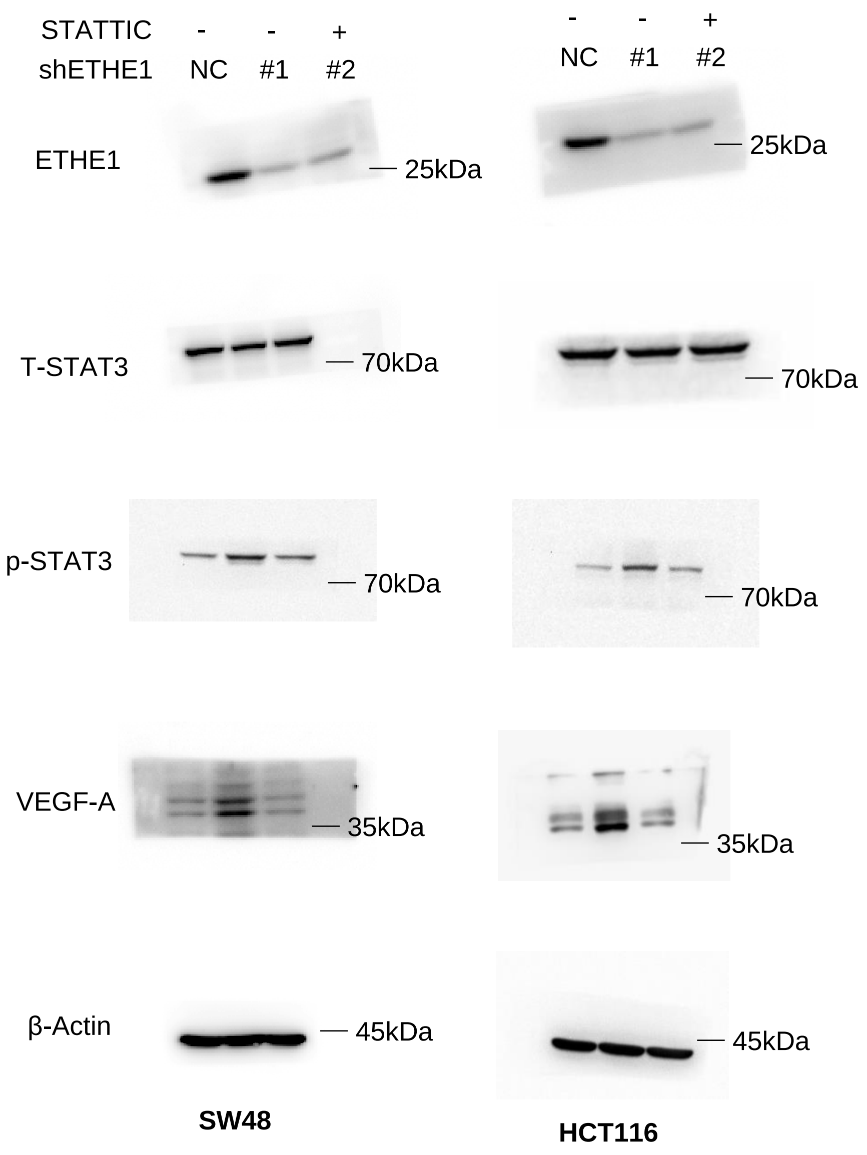
STATTIC - - +
shETHE1 NC #1 #2
- - +
NC #1 #2
ETHE1
25kDa
25kDa
T-STAT3
70kDa
70kDa
p-STAT3
70kDa
70kDa
VEGF-A
35kDa
35kDa
β-Actin
45kDa
45kDa
SW48
HCT116

Figure.6A

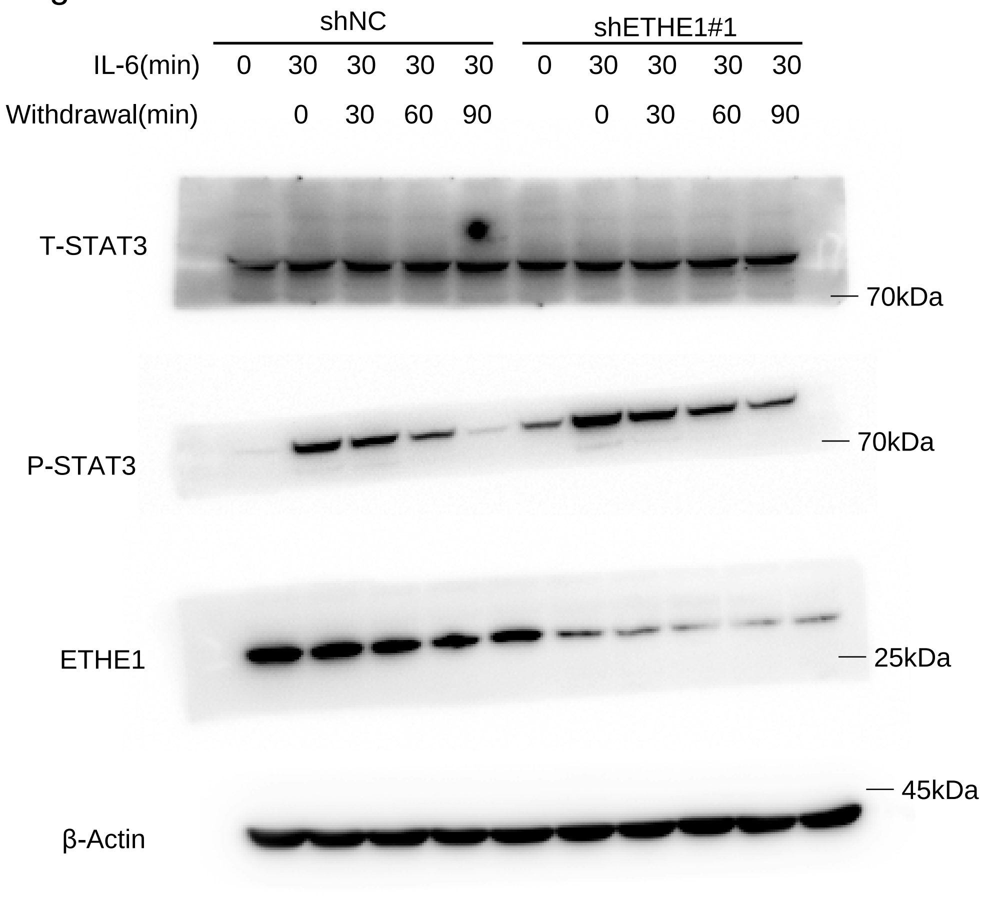

SW48

Figure.6B

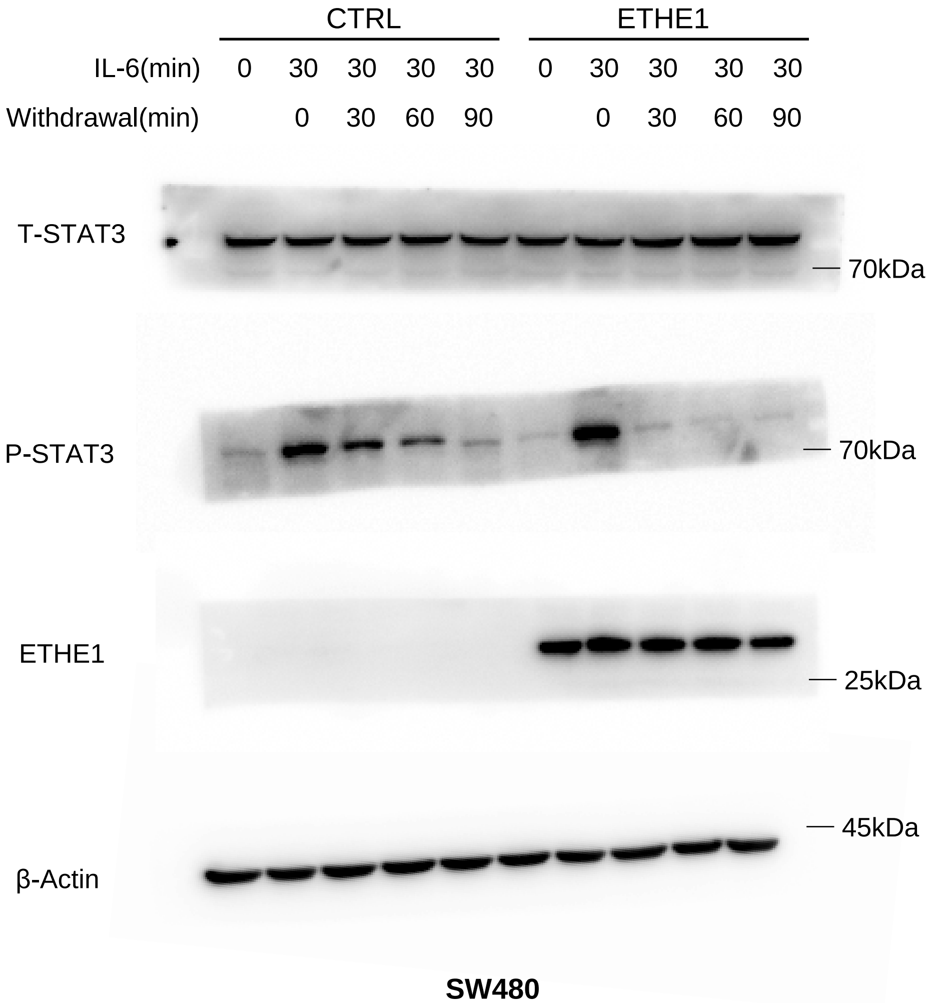

SW480

Figure.6C

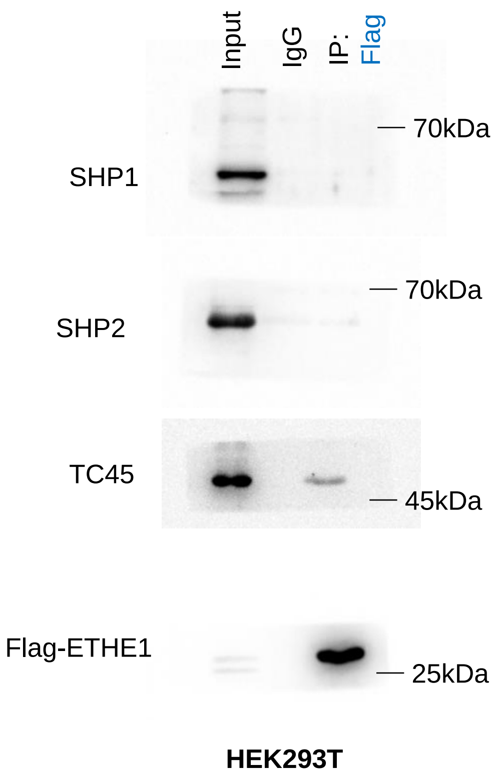

Figure.6D

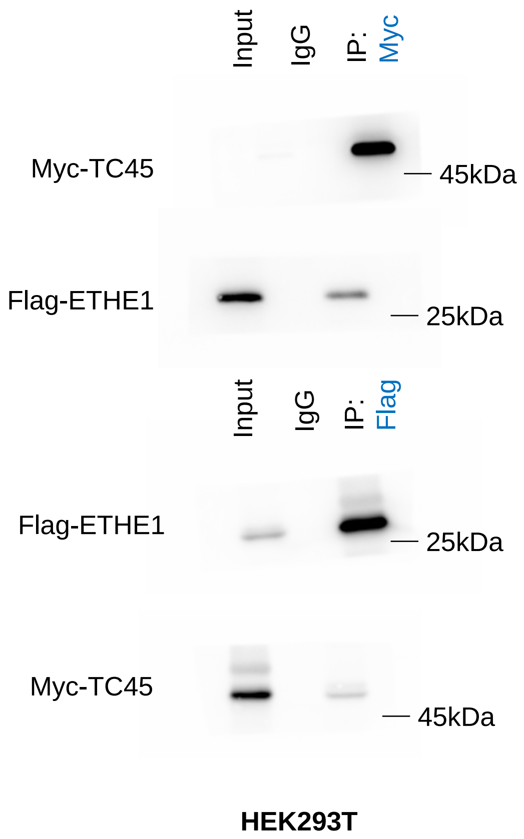

Figure.6E

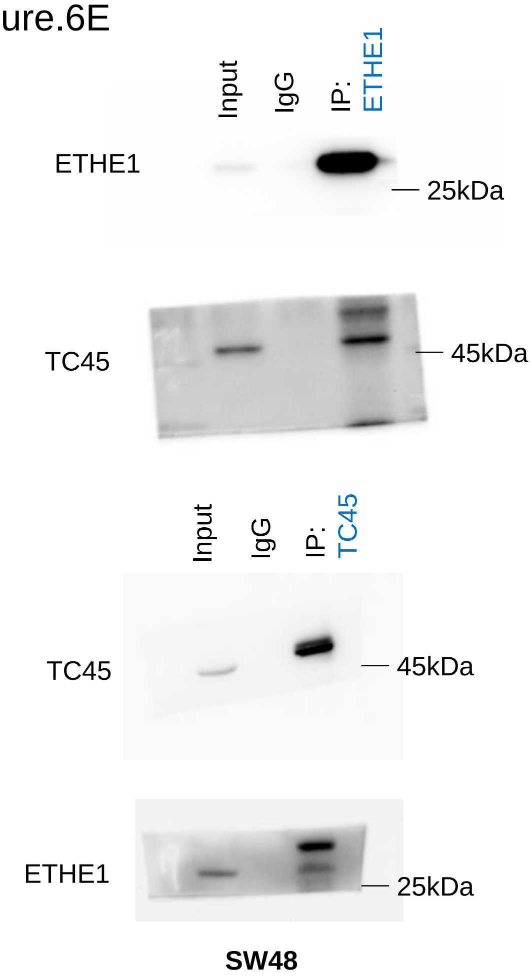

Figure.6F

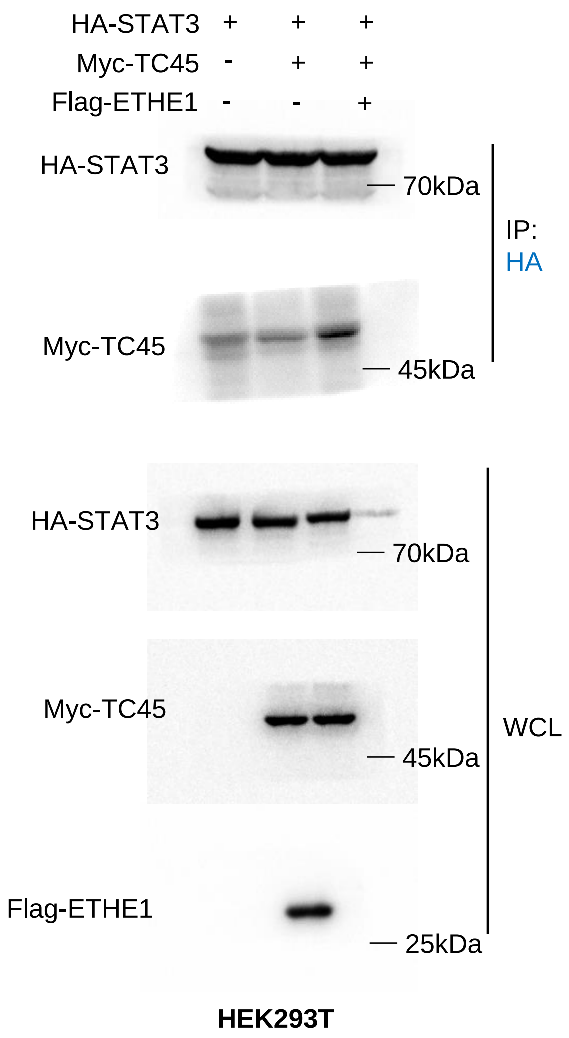

Figure.6G

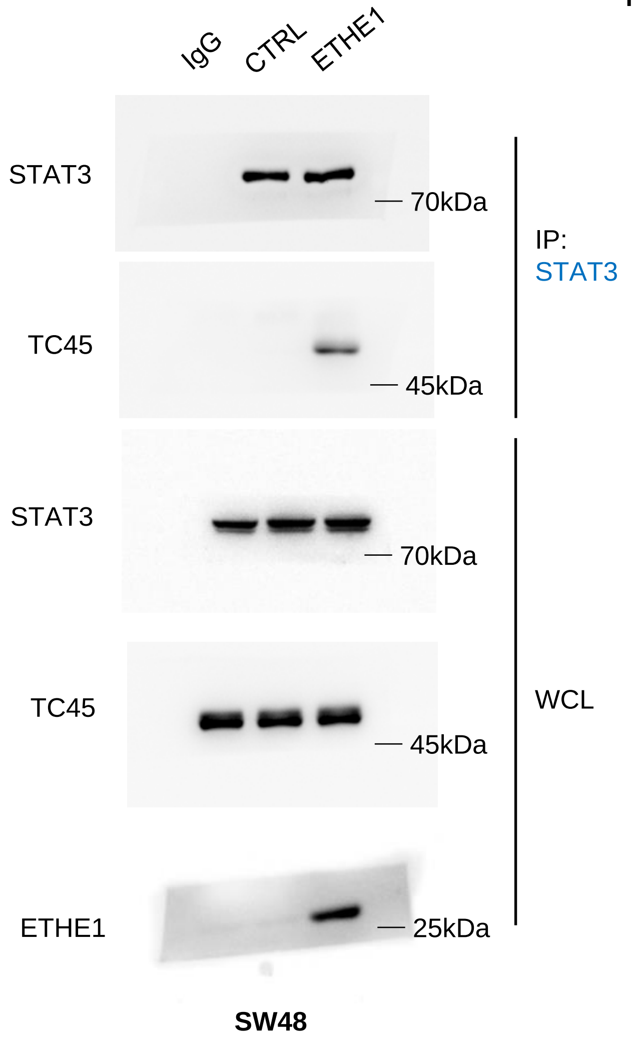

Figure.6H

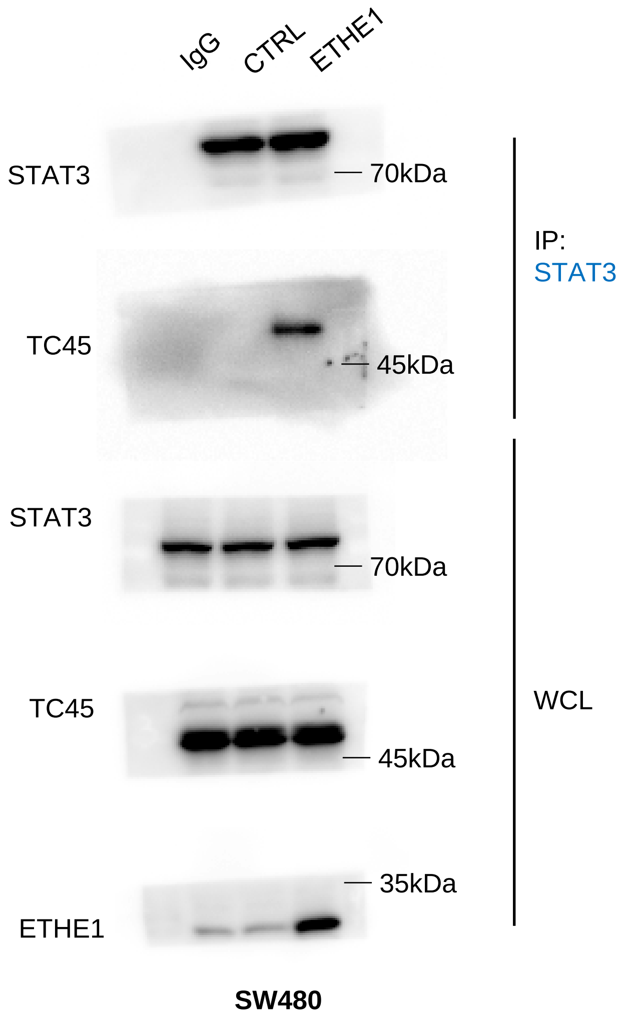

Figure.6J

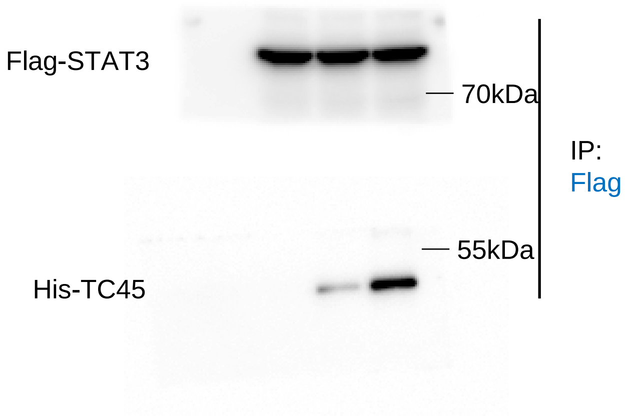

Figure.6J

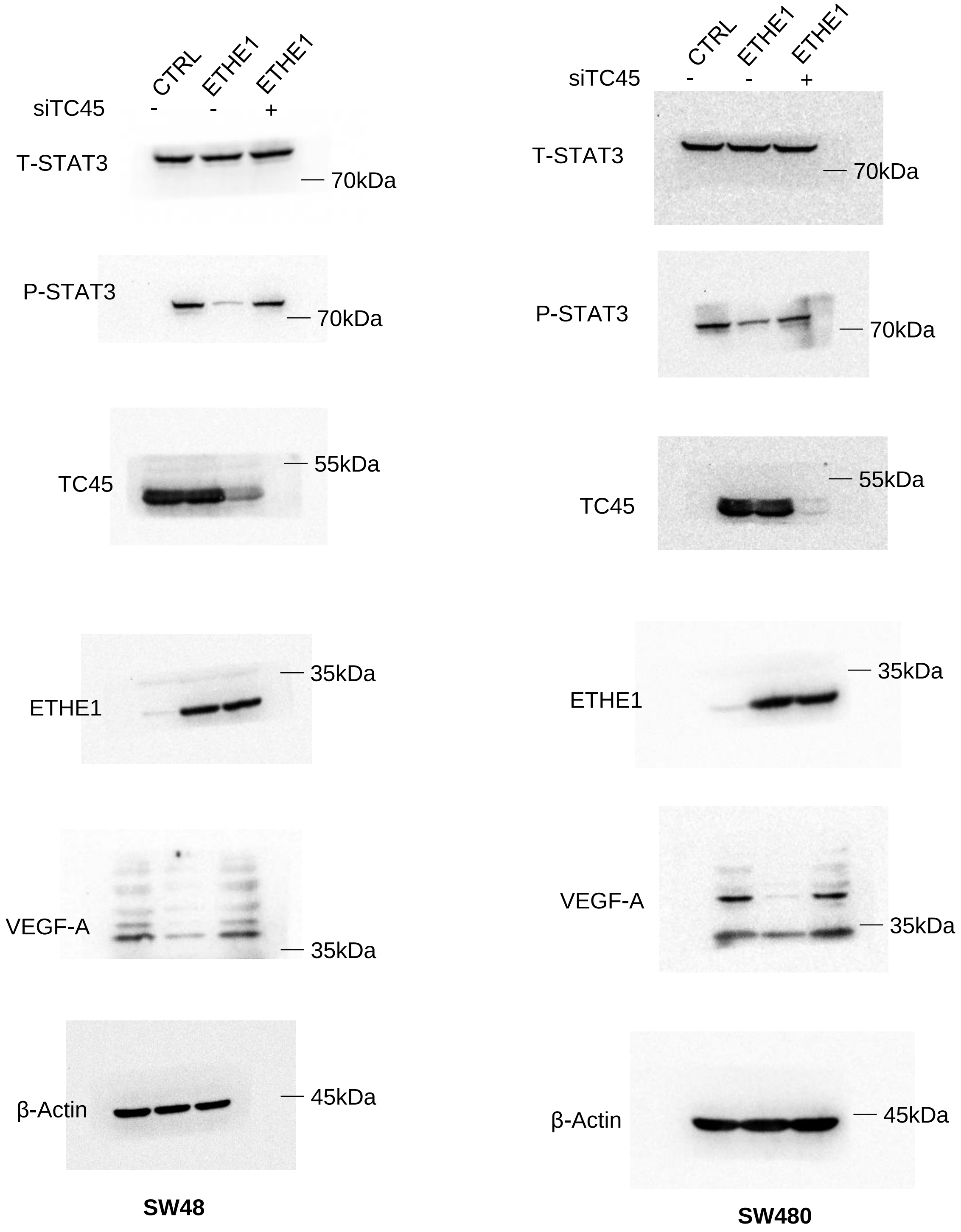

CTRL
ETHE1
ETHE1
siTC45
-
-
+
T-STATAT3
70kDa
P-STAT3
70kDa
TC45
55kDa
ETHE1
35kDa
VEGF-A
35kDa
β-Actin
45kDa
SW48
CTRL
ETHE1
ETHE1
siTC45
-
-
+
T-STAT3
70kDa
P-STAT3
70kDa
TC45
55kDa
ETHE1
35kDa
VEGF-A
35kDa
β-Actin
45kDa
SW480

Supplementary Figure. 1C

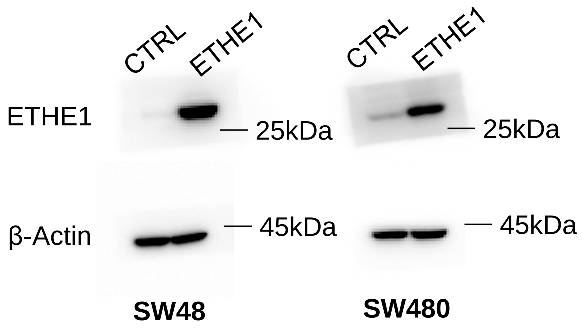

Supplementary Figure. 1D

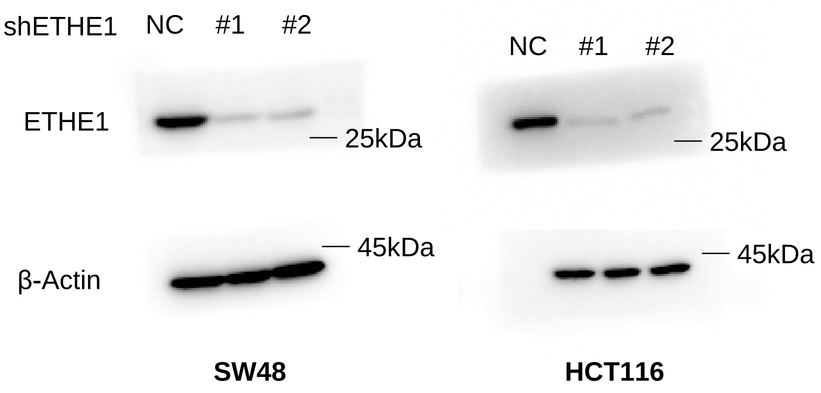

Supplement: Supplementary file 2 — Original Data File [file 41419_2024_7021_MOESM2_ESM.pdf]
